# Population Genomics and Connectivity of the Blue Mussel Species Complex: Insights From a North‐East Atlantic Hybrid Zone

**DOI:** 10.1111/eva.70185

**Published:** 2025-11-27

**Authors:** Eleonora Cariolato, Thomas Reed, Deirdre Brophy, Conor T. Graham, Frances E. Lucy, Luca Mirimin

**Affiliations:** ^1^ Department of Natural Resources & the Environment, School of Science and Computing, Marine and Freshwater Research Centre Atlantic Technological University Galway Ireland; ^2^ School of Biological, Earth and Environmental Sciences University College Cork Cork Ireland; ^3^ Centre for Environmental Research Innovation and Sustainability (CERIS) Atlantic Technological University, Sligo Sligo Ireland

**Keywords:** aquaculture, connectivity, *Mytilus*, North‐east Atlantic, population structure, SNPs

## Abstract

Blue mussels (*Mytilus* spp.) are ecologically and economically important bivalves widespread in both hemispheres. Their relevance to coastal ecosystems and the aquaculture industry has made them extensively studied. The *Mytilus* complex consists of distinct genetic lineages, including *Mytilus edulis* , *Mytilus galloprovincialis* , *Mytilus trossulus* , and their fertile hybrids. In overlapping areas, they create complex hybrid zones, which have been investigated along European coasts, employing multi‐marker approaches. However, knowledge gaps still exist in the North‐east Atlantic region, in the middle of their hybrid zone around the island of Ireland, regarding their genomic composition, population structure and connectivity. This study addresses these gaps by genotyping 781 individuals from 26 sites encompassing Ireland's hybrid zone, including both wild and farmed stocks from varying environmental conditions. Using a selected panel of 72 SNP markers we examined relationships among genotypic composition, genetic diversity, isolation by distance (IBD) and environmental variables to identify drivers of *Mytilus* genetic structure. Results confirmed two distinct genetic lineages and their hybrids, with a clear geographic pattern: the east coast of Ireland is dominated by pure *M. edulis* genotype populations, while the south, west and north coasts exhibit varying degrees of admixture with *M. galloprovincialis* genotype. Pure *M. galloprovincialis* populations were identified at specific sites on the west and north coast. Sea current resistance and wave height were significant predictors for both genotype composition and genetic differentiation. This study corroborates previous findings and provides the first comprehensive investigation of Irish *Mytilus* spp. population structure and connectivity using a multi‐marker approach. The findings highlight the importance of understanding the *Mytilus* complex's composition and population dynamics to inform sustainable aquaculture practices and monitor potential climate change‐driven shifts in the North‐east Atlantic region.

## 1 Introduction

The blue mussel (*Mytilus* spp.) is an economically and ecologically important bivalve, commonly distributed both in the northern and southern hemispheres (Gardner et al. 2021; Gosling 2021; Larraín et al. 2018; Mathiesen et al. 2017). It is often described as an ecosystem engineer and environmental indicator, providing several important ecosystem services (Barrett et al. 2022; van der Schatte Olivier et al. 2020). Moreover, blue mussels are an important species in the shellfish aquaculture industry worldwide (FAO 2024). In 2022, European aquaculture production yielded around 1.1 million tonnes of aquatic organisms worth €4.8 billion, of which mussels production accounted for more than 35% by weight and almost 10% by value (Eurostat, Aquaculture Statistics retrieved 6th February 2025). In northern Europe, mussel farming mostly relies on two culturing techniques: seabed culture and rope culture (Avdelas et al. 2021). Notwithstanding the effects of dredging and the addition of physical structures to promote settlement, mussel farming has low environmental impacts relative to other food production systems, making this aquaculture sector particularly sustainable and providing high‐quality animal proteins rich in Omega‐3 fatty acids (Avdelas et al. 2021; Barrett et al. 2022; Cooney et al. 2025; Yaghubi et al. 2021).

In the temperate Northern Hemisphere, the blue mussel complex comprises three congeneric species: *Mytilus edulis* (Linnaeus 1758), *Mytilus galloprovincialis* (Lamarck 1819) and *Mytilus trossulus* (Gould 1850). *M. edulis* , commonly referred to as the Atlantic blue mussel, is a cold‐temperate species with a distribution range that covers the eastern and western coasts of the North Atlantic, and the coasts of northern Europe up to the Arctic region (Beaumont et al. 2008; Bierne et al. 2003; Diz and Skibinski 2024; Gosling et al. 2008; Mathiesen et al. 2017; Nascimento‐Schulze et al. 2023; Simon et al. 2021). *M. galloprovincialis* , often referred to as the Mediterranean mussel, has a distribution range from the Black Sea, through the Mediterranean Sea to the North‐east Atlantic up to the Arctic region (Bierne et al. 2003; Gosling et al. 2008; Kijewski et al. 2011; Mathiesen et al. 2017; Vendrami et al. 2020). It is divided into two main lineages: the Atlantic and the Mediterranean (Bierne et al. 2003; del Rio‐Lavín et al. 2022; Kijewski et al. 2011; Lynch et al. 2020; Mathiesen et al. 2017; Simon et al. 2021; Vendrami et al. 2020). Moreover, *M. galloprovincialis* is also present in both the southern and northern hemispheres, on the Pacific and Atlantic coasts, highlighting its great capacity to settle and establish in regions outside its original distribution range (del Rio‐Lavín et al. 2022; Gardner and Westfall 2012; Larraín et al. 2018; Nascimento‐Schulze et al. 2023; Zbawicka et al. 2022). Finally, *M. trossulus* is originally from the Pacific Ocean, and it can be found on North Atlantic coasts, in the Baltic Sea and in the Arctic (Braby and Somero 2006; Dias, Piertney, et al. 2011; Mathiesen et al. 2017; Nascimento‐Schulze et al. 2023; Väinölä and Strelkov 2011; Vendrami et al. 2020). These three species are morphologically difficult to distinguish, making their population structure complex to investigate, especially when their distribution ranges overlap (Gosling and Wilkina 1981; Seed 1974).

In these overlapping areas, different lineages of blue mussels hybridise extensively, producing fertile offspring and a composite genomic make‐up, with varying levels of introgression and backcrossing with local populations (Bierne et al. 2003; Gosling et al. 2008; Vendrami et al. 2020). Their hybrid zones have been extensively studied in recent decades, with particular interest in the evolutionary mechanisms of speciation, adaptation and the impact of hybridisation on the aquaculture industry (Bierne et al. 2003; Dias, Malgrange, et al. 2011; Fraïsse et al. 2014; Michalek et al. 2016; Simon et al. 2021). In Europe, a well‐established hybrid zone between *M. edulis* and *M. galloprovincialis* extends from the north coast of France and south‐west England to the southwest and west of Ireland (Bierne et al. 2003; Coghlan and Gosling 2007; Diz and Skibinski 2024; Hilbish et al. 2002; Simon et al. 2019). Hybrid zones between *M. edulis* , *M. galloprovincialis* and *M. trossulus* are mostly present in northern European coasts in Scotland, the Baltic Sea, North Sea, Norwegian Sea, parts of the Barents and White Seas, and up to the Arctic Ocean in Greenland (Beaumont et al. 2008; Dias, Piertney, et al. 2011; Mathiesen et al. 2017; Väinölä and Strelkov 2011). Local environmental conditions such as temperature, salinity and wave exposure have been proposed as key drivers of *Mytilus* spp. local population structure (Bierne, David, Boudry, and Bonhomme 2002; Coghlan and Gosling 2007; Diz and Skibinski 2024; Gosling and Wilkina 1981; Hilbish et al. 2002; Lynch et al. 2020).

Genetic structure in the Northern Europe hybrid zones is temporally dynamic, with frequencies of pure types and their hybrids varying throughout the decades (Fly et al. 2015; Lynch et al. 2020). Several studies suggested that local environmental conditions and ongoing climate change could play a key role in these dynamics, via both pre‐zygotic mechanisms (e.g., effects on reproductive) and post‐zygotic mechanisms (e.g., differential effects on hybrid versus pure type survival, especially at the spat stage) (Beaumont et al. 2004; Doherty et al. 2009; Kenchington et al. 2020; Lynch et al. 2020; Shields et al. 2008). Climate change effects in the North‐east Atlantic region have been observed in the past few decades, with rising sea surface temperatures impacting the geographic distribution of cold‐water species (ICES 2024). However, this warming trend is interrupted by periodic cool spells and increased storminess, which contribute to greater freshening of coastal waters (ICES 2024; Nolan et al. 2023). Moreover, coastal currents play a central role in the connectivity and settlement of mussels and changes in these coastal currents can have a great impact on the shellfish industry (Avdelas et al. 2021; Demmer, Neill, et al. 2022; Demmer, Robins, et al. 2022).

A general decline in mussel production and the unreliability of natural spat supply (e.g., scarcity of natural mussel beds) have been observed around all European coasts since the 1990s, especially in Northern countries (Avdelas et al. 2021). According to Baden et al. (2021), in the past few decades, mussel beds in the North‐east Atlantic sheltered littoral and sublittoral zones have declined by more than 50%. In most areas, the decline is generally attributed to poor recruitment success, often linked to the increasing frequency of extreme weather events driven by climate change (Avdelas et al. 2021; Baden et al. 2021; Little et al. 2024). In France, recurrent mass mortalities in hybrid zones between *M. edulis* and *M. galloprovincialis* have been observed by Benabdelmouna and Ledu (2016), who hypothesised cytogenic anomalies linked to hybridisation as a potential cause. Moreover, in the review by Lupo et al. (2021), a higher mortality risk between hybrids of *M. edulis* and *M. galloprovincialis* compared to pure strains was reported, as well as a link with lower heterozygosity of *M. edulis* . Given the decline in wild and farmed mussel stocks, the environmental pressures of climate change, and the complexity of mussel population structure and hybridisation dynamics, an in‐depth investigation of mussel population genomics and connectivity in the North‐east Atlantic and northern Europe is essential to establish a baseline for future monitoring.

In the past 10 years, new tools and methodologies have been developed to investigate the population structure of the blue mussel complex (Fraïsse et al. 2016; Mathiesen et al. 2017; Nascimento‐Schulze et al. 2023; Simon et al. 2019; Wilson et al. 2018). The advent of new and increasingly affordable multi‐marker approaches, such as single nucleotide polymorphisms (SNPs), has allowed more powerful and comprehensive analyses of species and population genetics, especially for investigating the level of hybridisation and introgression in hybrid zones (Mathiesen et al. 2017; Simon et al. 2019), compared to the traditional nuclear single marker approach (e.g., *Me15*/*Me16* primers; Inoue et al. 1995). These advancements have not only increased the availability of genetic data but also enhanced the integration of genetic information with other data types (e.g., environmental variables) (Wenne et al. 2020, 2022) and increased the scope for investigating population structure, phylogeography and the adaptive potential in the context of seascape genomics.

Despite the growing body of genomic research on *Mytilus* spp., Ireland remains underrepresented in many large‐scale studies (Nascimento‐Schulze et al. 2023; Vendrami et al. 2020). This is notable given that Ireland includes an extensive hybrid zone and represents an ecologically and economically important region within the North‐east Atlantic. Although the Irish hybrid zone has been monitored since the 1970s, the increasing impacts of climate change, combined with a growing mussel aquaculture sector that has recently faced severe declines in output and revenue, call for a more fine‐scale and genomically informed investigation (Bord Iascaigh Mhara 2024; Gosling et al. 2008; Lynch et al. 2020; Seed 1974). While several Northern European countries have adopted SNP‐based and multi‐locus approaches, research in Irish waters still relies largely on single‐marker screening (Gosling et al. 2008; Lynch et al. 2020). As such, Ireland represents a relevant case study to provide additional baseline genomic data on blue mussel populations, supporting broader efforts to understand connectivity and hybridisation in the North‐east Atlantic.

Thus, the aims of this study are to use a panel of SNP markers originally sourced from selected publications addressing genetic structure at the inter‐ and intraspecific levels of the *Mytilus* spp. complex in European waters (Fraïsse et al. 2016; Hammel et al. 2021; Simon et al. 2018; Wilson et al. 2018), along with a geographically comprehensive sampling across the island of Ireland as a case study to: (i) elucidate the genetic composition and population structure of the *Mytilus* spp. complex in the Celtic and Irish Seas, including admixed and pure *M. edulis* populations; (ii) investigate the distribution of the different genotypes around the Irish coasts; and (iii) explore oceanographic and environmental factors that could drive the observed population structure, as insight into potential connectivity, here defined as the inferred potential for gene flow between sites, estimated through patterns of ocean current resistance and genetic differentiation.

## 2 Materials and Methods

### 2.1 Study Sites and Sampling

**FIGURE 1:**
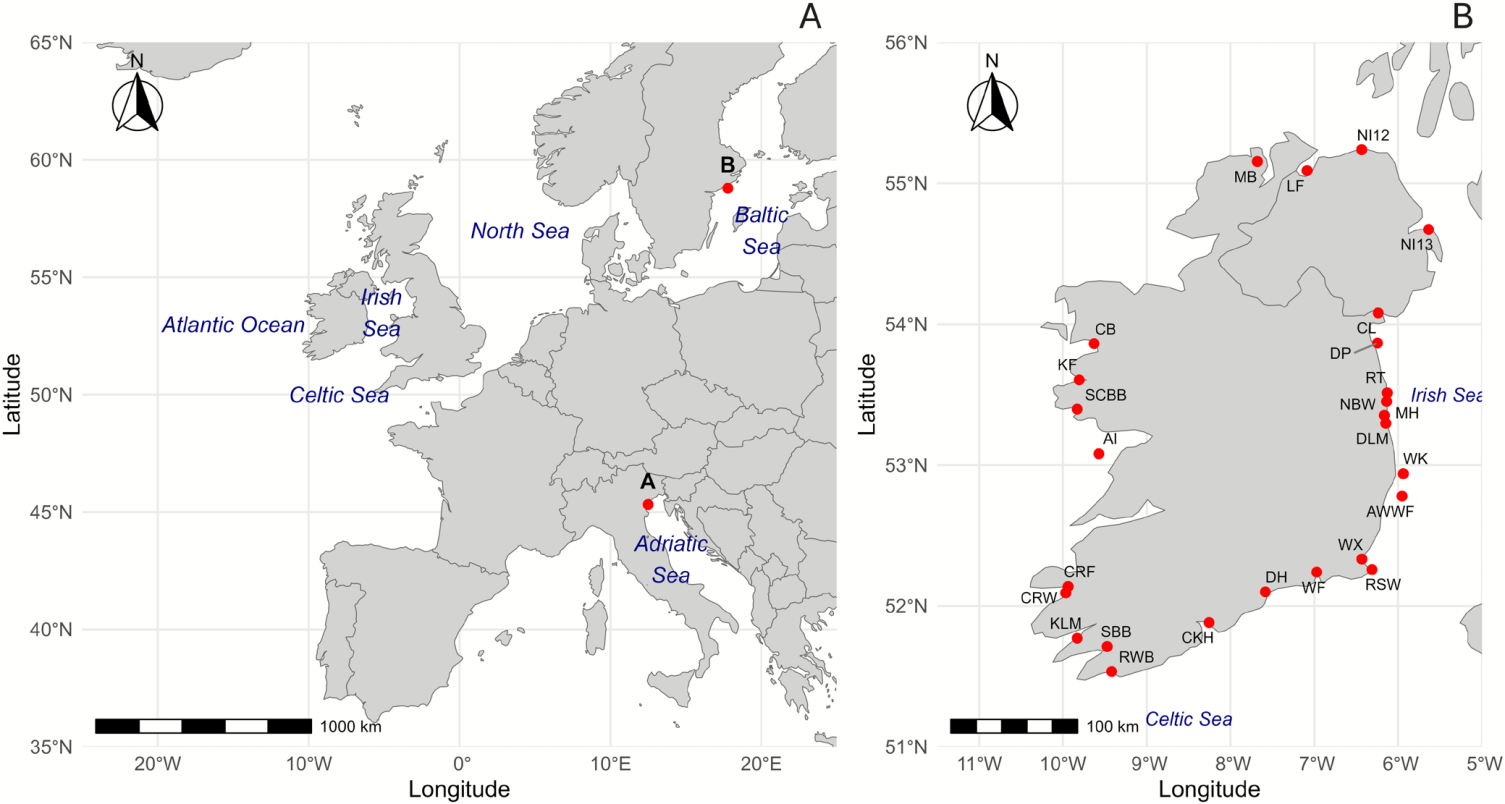
Mussel sampling locations. (A) Ireland within the European context, showing the Celtic Sea, Irish Sea, North Sea, the Atlantic Ocean, as well as the Adriatic and Baltic sample locations. (B) Zoomed‐in map of Irish sampling sites. Location codes correspond to Table 1.

A total of 781 adult individuals of *Mytilus* spp. (shell length between 45 and 55 mm) were sampled in 26 locations around Ireland (Figure 1), from a variety of habitats and stock types (i.e., farmed and wild stocks). Samples were collected by local stakeholders either by hand, harvested from ropes by mussel workboat, or by dredging, and shipped fresh to the Atlantic Technological University (ATU)—Galway city and stored at −20°C. In addition, mussel tissues preserved in absolute ethanol from the Adriatic Sea (Italy, *n* = 11) and from the Baltic Sea (Sweden, *n* = 10) were included as reference/outlier samples, which represent other lineages present in Europe but not in Irish waters. Sampling details are summarised in Table 1.

**TABLE 1:** Information on the sampling campaign including: Sampling locations, geographic coordinates, sampling month and year, location code, number of individuals screened per population (*N*), stock type, habitat type, type of culture (in case of farm stock) or type of substrate (in case of wild stock).

Country	Location	Region	Latitude	Longitude	Sampling date	Code	*N*	Stock type	Habitat	Type of culture/substrate
Republic of Ireland	Mulroy Bay	North	55.15488	−7.68000	November 2022	MB	30	Farm	Subtidal	Rope growth
	Lough Foyle	North	55.09047	−7.08548	November 2022	LF	30	Farm	Subtidal	Bottom growth—seabed
	Clew Bay	West	53.86222	−9.62820	April 2022	CB	29	Farm	Subtidal	Rope growth
	Killary Fjord	West	53.60487	−9.80624	June 2022	KF	29	Farm	Subtidal	Rope growth
	Bertraghboy Bay (South Connemara)	West	53.39689	−9.82947	February 2023	SCBB	22	Wild	Intertidal	Natural rock
	Aran islands (Inish Meáin)	West	53.08083	−9.57142	July 2022	AI	30	Wild	Intertidal	Artificial structure
	Cromane Wild	Southwest	52.09275	−9.96550	June 2022	CRW	30	Wild	Subtidal	Bottom growth—seabed
	Cromane Farm	Southwest	52.13825	−9.93817	June 2022	CRF	30	Farm	Intertidal	Bottom growth—seabed
	Snave Bantry Bay	Southwest	51.71223	−9.47268	November 2022	SBB	30	Farm	Subtidal	Rope growth
	Roaringwater Bay	South	51.53507	−9.41850	June 2022	RWB	28	Farm	Subtidal	Rope growth
	Cork Harbour	South	51.88000	−8.25000	March 2023	CKH	30	Wild	Intertidal	Bottom growth—seabed
	Waterford Estuary	South	52.24000	−6.97000	October 2022	WF	30	Wild	Intertidal	Bottom growth—seabed
	Kilmakilloge	South	51.77100	−9.82938	June 2022	KLM	30	Farm	Subtidal	Rope growth
	Dungarvan Harbour	South	52.10006	−7.58435	December 2022	DH	29	Wild	Intertidal	Bottom growth—seabed
	Rosslare	East	52.25820	−6.31087	July 2022	RSW	30	Wild	Subtidal	Bottom growth—seabed
	Wexford Harbour	East	52.33273	−6.43135	July 2022	WX	30	Farm	Subtidal	Bottom growth—seabed
	Arklow Wind Farm	East	52.78000	−5.95231	July 2023	AWWF	30	Wild	Intertidal	Artificial structure
	Wicklow	East	52.93878	−5.93775	May 2022	WK	30	Wild	Subtidal	Bottom growth—seabed
	Dún Laoghaire Marina	East	53.29000	−6.14000	September 2022	DLM	30	Wild	Intertidal	Artificial structure
	North Bull wall	East	53.35000	−6.16000	September 2022	NBW	30	Wild	Intertidal	Bottom growth—seabed
	Malahide	East	53.45000	−6.13000	September 2022	MH	30	Wild	Intertidal	Bottom growth—seabed
	Rogerstown	East	53.51256	−6.13003	November 2022	RT	30	Wild	Intertidal	Bottom growth—seabed
	Dunany Point	East	53.86600	−6.24622	November 2022	DP	30	Wild	Intertidal	Bottom growth—seabed
	Carlingford Lough	East	54.07942	−6.23708	May 2022	CL	30	Farm	Subtidal	Bottom growth—seabed
Northern Ireland (UK)	Dunseverick	North	55.23881	−6.43339	August 2023	NI‐12	35	Wild	Intertidal	Rocky shore
	Bangor	North	54.67210	−5.63544	September 2023	NI‐13	39	Wild	Intertidal	Rocky shore
Italy	Piattaforma acqua alta	Adriatic Sea	45.31667	12.50000	May 2022	A	11	Wild	Intertidal	Artificial structure
Sweden	Sundsbådan	Baltic Sea	58.78970	17.77921	September 2021	B	9 Wild		Subtidal	NA

*Note:* NA notation when information was not provided. See the map in Figure 1 for sampling locations.

### 2.2 DNA Extraction

Gill tissue was dissected from thawed *Mytilus* samples and preserved in absolute ethanol to establish a tissue bank (currently stored and available upon request at the Marine and Freshwater Research Centre in ATU‐Galway, Republic of Ireland). DNA extraction was carried out using the E.Z.N.A. Tissue kit (Omega BioTek, Norcross, GA, USA) and according to the manufacturer's instructions with adaptations: to homogenize the tissue, in a nuclease‐free 1.5 mL microcentrifuge tube, approximately 30 mg of gill tissue was minced by vortex at maximum speed for 15 min with three Zirconium Ceramic Oxide beads (diameter 1.4 mm, Fisherbrand). Then, incubation at 55°C was performed for 2 h with vortexing every 30 min for 15 s.

To ensure that the protocol yielded sufficient amounts of DNA for downstream genotyping procedures, genomic DNA of a subset of samples was quantified with the dsDNA Broad Range Assay kit using a Qubit 3.0 fluorometer (Thermofisher Scientific, Ireland).

### 2.3 Adhesive Protein Gene Single Marker Genotyping

To confirm successful DNA extraction and to obtain a preliminary genetic/taxonomic screen in line with previous studies carried out in Ireland (Gosling et al. 2008; Lynch et al. 2020), all samples were genotyped using a single marker approach developed by Inoue et al. (1995) that targets the foot adhesive protein gene with primers *Me15* and *Me16*. PCR was carried out in a 20 μL reaction mixture containing: AccuStart II PCR ToughMix (1×) (Quantabio), GelTrack Loading Dye (1×) (Quantabio), 0.5 μM of primer *Me15* (5′‐CCAGTATACAAACCTGTGAAGA‐3′), 0.5 μM of primer *Me16* (5′‐TGTTGTCTTAATGGTTTGTAAGA‐3′), and 2 μL of template DNA (concentration between 2 and 40 ng/μL). The thermal conditions were as follows: 94°C for 3 min followed by 35 cycles of [95°C for 15 s, 56°C for 20 s, 72°C for 20 s]. The PCR was carried out using a MiniAmp Plus thermocycler (Applied Biosystem). The visualisation of the amplicons was conducted through gel electrophoresis in a 2% agarose gel stained with SYBR Safe DNA Gel Stain (Invitrogen, CA, USA), run at 100 V for 50 min. The size of PCR amplicons was established by comparison to a GeneRuler 50 bp DNA Ladder ready‐to‐use (Thermo Scientific, Ireland) upon visualisation by UV light in a Bio‐Rad Gel Doc EZ Imager (Bio‐Rad, Ireland).

### 2.4 SNPs Selection and Genotyping

The set of SNPs used in the present study was originally sourced from selected publications addressing genetic structure at the inter‐ and intraspecific levels of the *Mytilus* spp. complex in European waters (Fraïsse et al. 2016; Hammel et al. 2021; Simon et al. 2018; Wilson et al. 2018), aiming for discrimination between pure lineages and hybrids found in Irish waters, as well as cost‐effectiveness. To facilitate genotyping using a Biomark HD system (Standard BioTools, South San Francisco, CA, USA), primer pairs were designed using 1000 bp flanking sequences from the *M. galloprovincialis* genome assembly (LOLA—European Nucleotide Archive, project IDs PRJEB24883; Gene Bank GCA_900618805.1; Gerdol et al. 2020), whereby conserved DNA regions were identified by alignment against 17 additional *Mytilus* genomes (Corrochano‐Fraile et al. 2022; Gerdol et al. 2020; Murgarella et al. 2016; Regan et al. 2024; M. Gerdol, personal communication). Ultimately, a panel of 91 SNPs that showed a clear genotype distinction across reference genomes, and flanking regions suitable for primer design was retained for genotyping the study samples, after a pre‐amplification step: 70 SNPs from Simon et al. (2018) and 9 from Hammel et al. (2021) (all of which were originally published in Fraïsse et al. 2016), and 12 from Wilson et al. (2018). Data from each run were analysed using the Standard BioTools SNP Genotyping Analysis software v.1.0.2 (Standard BioTools, https://www.standardbio.com/), with an assay reference library. Within each run, the optimal cycle for each SNP was determined based on cluster segregation and amplification success. All base calls were manually verified and adjusted to ensure the accurate identification of homozygous and heterozygous clusters. SNPs that showed either undefined cluster patterns or amplification failure in more than 15% of the samples were discarded. Similarly, samples that failed to amplify more than 15% of the SNP markers were discarded from the dataset. We applied this 15% threshold as a more stringent criterion compared to other studies (Mathiesen et al. 2017; 25%), to reduce missing data and increase the reliability of downstream analyses.

Details on primer design, reference genomes and protocol optimisation are provided in Supporting Information.

### 2.5 Descriptive and Summary Statistics

An explorative and diagnostic analysis of the full dataset was performed, including 26 Irish sites, 1 Adriatic site and 1 Baltic site. The R package *Adegenet* (Jombart 2008; Jombart and Ahmed 2011; R Core Team 2023) was used to run a Discriminant Analysis of Principal Components (DAPC) and customisation from the Rscript ‘ggDAPC’ (Frantine 2023; GitHub—https://github.com/wilsonfrantine/ggDAPC) was used to produce all the DAPC plots of this study. The function *sNMF* of the package *LEA* (Frichot and François 2015) was used to run several admixture analyses with different numbers of predefined clusters (*K*), which ranged from two to five, to explore the population structure and admixture of the different populations. To assess the quality of the markers, allele frequencies were calculated for each locus within each population to identify monomorphic markers, as well as the inbreeding coefficient *F*_is_ per locus across populations, within populations and per population across loci using the R package *Genepop v.1.2.2* (Rousset 2008). Loci that were either monomorphic or with minor allele frequencies < 0.01 were discarded after manual inspection (McDevitt et al. 2022). Loci with a global *F*_is_ > 0.3 or < −0.3, indicating departures from Hardy–Weinberg expectations, were further examined population by population, to determine whether high absolute values for *F*_is_ could be explained by admixture. Loci that showed a *F*_is_ > 0.3 or < −0.3 in more than 50% of the populations, excluding those that displayed a consistent level of admixture (based on the admixture preliminary analysis and population‐level *F*_is_), were discarded from downstream analyses. The threshold of |0.3| was chosen empirically, based on the distribution of *F*_is_ values across loci in this dataset, and was used as a conservative quality control filter to minimise the risk of including loci with technical artefacts or extreme genotype frequency distortions (heterozygote deficiencies or excesses). After this step, 72 SNPs were retained for the subsequent analyses (list of SNPs loci dataset in Table S1).

### 2.6 Population Structure of Irish *Mytilus* spp.

A reduced dataset including only samples from the Irish coast was used to assess the genetic makeup and population structure of Irish mussels. A DAPC was run (as detailed above), and allele richness (*A*_r_), observed heterozygosity (*H*_o_) and expected heterozygosity (*H*_e_) for each population were calculated with the function *divBasic* from the package *diveRsity* (Keenan et al. 2013). The R packages *RLDNe* (Do et al. 2014; Robinson 2024), *dartRverse* and *dartR* (Gruber et al. 2018; Mijangos et al. 2022) were used to calculate the effective population size (*N*_e_) employing the function *gl.LDNe*. STRUCTURE v2.3.4 (Pritchard et al. 2000) was employed to analyse the genetic structure and the putative number of clusters of the Irish populations. The analysis parameters were as follows: 5000 length of burn‐in period, 50,000 MCMC, admixture model, allele frequencies correlated, *K* from 1 to 8, with 5 repetitions per *K*. The results were uploaded to the CLUMPAK online server (Kopelman et al. 2015; https://clumpak.tau.ac.il/bestK.html, accessed: 5th October 2024), and the best *K* was chosen following the Evanno method (Evanno et al. 2005). Furthermore, the software FSTAT V2.9.4 (Goudet 2001) was used to calculate populations' pairwise differentiation index *F*_st_ with significance *p*‐value corrected by Bonferroni multiple testing. Parameters were set as follows: global test of Hardy–Weinberg within and overall samples (500 iterations), ‘population differentiation’ test not assuming HW within samples, 5/100 nominal level for multiple tests and 1000 permutations. To minimise the influence of missing data on pairwise *F*_st_ with significance testing, we applied an additional filtering step. Loci with elevated missing genotypes (2%–8%) that interfered with *F*_st_ computation were iteratively inspected and discarded. Nine loci were therefore discarded, resulting in a final dataset of 63 loci used for the *F*_st_ analysis (Table S1).

All plots of this study were produced with the R packages *ggplot2* (Wickham et al. 2016) and *ggalluvial* (Brunson and Read 2023).

### 2.7 Admixed and *M. edulis* Populations in Irish Mussels

A further investigation was conducted on the Irish populations that showed a ‘pure’ Atlantic *M. edulis* genotype. Based on the STRUCTURE *Q* values from the Irish *Mytilus* spp. analysis, populations for which samples showed admixture proportions > 0.2 (Mathiesen et al. 2017) were categorised as ‘Admixed Irish Populations’, while populations for which samples showed admixture proportions < 0.2 were categorised as ‘Irish *M. galloprovincialis* genotype’ or ‘Irish *M. edulis* genotype’, depending on the dominant cluster, respectively.

To assess the population structure of Irish *M. edulis* , the ‘Irish *M. edulis* genotype’ populations were analysed with a separate DAPC and STRUCTURE with the same parameters set as above. Two datasets were analysed: (i) all Irish populations with an average admixed proportion < 0.2 and (ii) only Irish populations in which all individuals showed admixture < 0.2 (i.e., excluding populations in which only some individuals showed a higher level of admixture).

### 2.8 Isolation by Distance and Correlation Between Genotype Composition, Pairwise *F*_st_ and Environmental Variables

To investigate potential drivers of the genetic differentiation and genotype composition of the Irish mussels, a two‐step approach was used. First, the relationship between genotype composition (extent of admixture) and environmental variables was examined at each site. We then investigated if genetic differentiation between sites was related to the least‐cost distance between them, taking into account sea currents and environmental differences between sites.

#### 2.8.1 Environmental Variables Acquisition

The Regional Operational Model (ROMS) for the Northeast Atlantic was used to characterise the environmental conditions within the study area, which has a mean horizontal resolution of 1.9 km (Nagy et al. 2020; https://www.marine.ie/site‐area/data‐services/marine‐forecasts/ocean‐forecasts accessed on 11‐February‐2025). For each site, we obtained the long‐term minimum (min), maximum (max) and average (mean) of sea surface temperature (SST °C), salinity (PSU) for the period 2017–2023 and wave height (m) for the period 1994–2023. Some inshore sites fell outside of the area covered by the ROMS model. For these sites, data were extracted from the closest grid square for which data were available. The distance between the study sites and the geographic co‐ordinates of the modelled environmental data ranged from 144 m to 11.79 km, with a mean distance of 4.8 km (Table S4 and Figure S6).

#### 2.8.2 Oceanographic and Geographic Resistance to Dispersal

Resistance to dispersal between sites during the main dispersal period for *Mytilus* spp. (March–May) (Doherty et al. 2009) was estimated using mean eastward and northward sea surface current velocities (u‐component velocity and v‐component velocity, respectively, in m s^−1^) during those months for the period 2012–2023. Current data were obtained from the Northeast Atlantic ROMS model described above, and from the Global Ocean Physics Reanalysis, E.U. Copernicus Marine Service Information (CMEMS) *Marine Data Store* (MDS) (DOI: 10.48670/moi‐00021; accessed on 11‐February‐2025). Rasters of current data from the two data products were combined to create a resistance layer with the *disaggregate* and *merge* functions from the *raster* package in R (Hijmans 2025) preserving the higher spatial resolution of the Northeast Atlantic ROMS model.

In the *gdistance* package in R (van Etten 2017), a geographical correction was applied to the resulting resistance layer with the *geoCorrection* function and the least‐cost distance of dispersing between each pair of sites was calculated using the *costDistance* function. This produced an anisotropic matrix of least‐cost distance estimates (in arbitrary units) for each pair of sites, considering movement in both directions (i.e., from site ‘a’ to site ‘b’, and from site ‘b’ to site ‘a’).

For each pair of sites, a single least‐cost distance value was obtained using the minimum of the least‐cost distances from ‘a’ to ‘b’ and from ‘b’ to ‘a’. When dispersal between sites was not possible without moving against the direction of the currents or crossing land, the least‐cost distance estimate was equal to infinity. Of the 325 possible connections, 148 were calculable (i.e., the least‐cost distance was below infinity in at least one direction). For these 31 site pairs, the least‐cost paths did not reflect realistic transport scenarios (e.g., involved transport off the shelf edge for over 1000 km before deflection back towards the coast). We therefore grouped the least‐cost distance values into three categories representing the extent to which ocean currents presented a barrier to exchange between sites: cat0: least cost distance = infinity (strong current resistance), cat1: > 3,000,000 (moderate current resistance) and cat2: least cost distance < 1,100,000 (light current resistance).

To visualise the relative connectedness of sites based on ocean current resistance, the least‐cost distance values were plotted for site pairs in cat2 to produce a ‘sink‐source’ network map using the R packages *ggplot2* and *rnaturalearth* (Massicotte and South 2023).

For a detailed description of the construction of the resistance layer, the calculation of least‐cost distances, and the handling of land cells, please refer to Methods S2.

#### 2.8.3 Statistical Analyses

To investigate the potential contributions of environmental conditions to the genotype composition at each site, a beta regression model was run using the *betareg* R package (Cribari‐Neto and Zeileis 2010). The response variable was the STRUCTURE *Q* values for the *M. edulis* genotype for each site. A preliminary beta regression model was run including all the explanatory variables *Q*_valuesEdulis ~ mean_SST + max_SST + min_SST + mean_salinity + max_salinity + min_salinity + mean_significant_wave_height + max_significant_wave_height + min_significant_wave_height. Variance inflation factors were used to check for collinearity of the explanatory variables with the *vif* function from the *car* R package (Fox and Weisberg 2019). The best fitting beta regression model was selected with the *dredge* function from the *MuMIn* R package to perform automated model selection (Bartoń 2024), using subset to exclude collinear variable combinations (e.g., min, max and mean SST). To confirm that the selected model explained significantly more variance than an intercept‐only model, a likelihood ratio test (LRT) was performed by comparing the log‐likelihoods of the full and null models. Model diagnostics were performed by examining residuals and leverage values (hat values) using the base R function *hatvalues* (package *stats*). The model coefficients were expressed relative to the original scale of the response variable using the inverse logit transformation with the *inverse.logit* (Canty and Ripley 2022; Davison and Hinkley 1997). Model predictions were visualised using *ggplot2* and *ggeffects* (Lüdecke 2018) R packages.

To investigate the relationship between genetic divergence and ocean current resistance, a beta regression model was used in which pairwise *F*_st_ was the response variable and resistance category (factor3 levels) was the explanatory variable, using *betareg*. LRT and model residuals were also examined. Negative *F*_st_ values were replaced by 0.001 to meet beta regression assumptions (Wenne et al. 2022).

A post hoc testing of the estimation of marginal means was performed using the R package *emmeans* (Lenth 2025).

Finally, a more comprehensive beta regression model was run with pairwise *F*_st_ as the response variable, and current resistance category (factor with 3 levels), absolute difference in min SST (Δ°C), max salinity (ΔPSU) and max wave height (Δ*m*) between sites as explanatory variables. The analysis steps followed the same procedure described above, including selection of the best‐fitting model, LRT, diagnostic checks (residuals), and post hoc testing of estimation of marginal means.

## 3 Results

### 3.1 Descriptive and Summary Statistics

Preliminary single marker genotyping (Adhesive protein gene, Inoue et al. 1995) confirmed the expectations of the low‐resolution power compared to the multi‐marker approach (SNPs panel) by misidentifying 17% of *M. edulis* , 13.6% of *M. galloprovincialis* and 50.5% of mixed ancestry (Figure S1, data available upon request).

Explorative analyses based on DAPC and Admixture showed that samples from the Baltic and the Adriatic Seas were genetically distinct from the Irish populations, in line with the genetic lineages of blue mussels in those regions: *M. galloprovincialis* for the Adriatic Sea, and *M. trossulus* for the Baltic Sea around the Askö area (Stuckas et al. 2017; Vendrami et al. 2020). Furthermore, no evidence of *M. trossulus* genetic ancestry was detected along Irish coasts (Figures S2 and S3). However, the sample size of the Baltic population is small compared to the rest of this study, and further sampling in this region would be necessary to confidently assess the presence or absence of *M. trossulus* introgression in Ireland. Consequently, the Baltic and the Adriatic populations were excluded from the downstream analyses that focused exclusively on the Irish populations.

Allele frequencies calculated per locus within each population identified six SNP loci that were monomorphic for the Irish populations and the Adriatic population. Additionally, two loci that were not strictly monomorphic had a minor allele frequency < 0.01. These eight SNP loci were excluded from subsequent analysis.

Global *F*_is_ values calculated for each locus across all populations identified 16 loci with *F*_is_ values that were either > 0.3 or < −0.3, suggesting deviations from Hardy–Weinberg expectations. These 16 loci were then closely analysed at a population level; for 12 populations, more than 50% of this subset of loci had *F*_is_ values beyond the threshold. These 12 populations (Inish Meáin from the Aran Islands—hereafter Aran Islands—AI, Clew Bay—CB, Killary Fjord—KF, Mulroy Bay—MB, Snave Bantry Bay—SBB, Waterford Estuary—WF, Malahide—MH, Cork Harbour—CKH, Kilmakilloge—KLM, Dungarvan Harbour—DH, Bertraghboy Bay—SCBB and Roaringwater Bay—RWB) in fact showed a consistent level of admixture (see Figure S3 and Section 3.2).

To discard SNP loci with high or low *F*_is_ unrelated to admixture, global loci *F*_is_ were recalculated excluding these 12 admixed populations (i.e., mixed ancestry from both genetic backgrounds for > 20% Mathiesen et al. 2017). Ultimately, three loci for which the *F*_is_ values exceeded the |0.3| threshold for more than 50% of the non‐admixed populations were removed, resulting in a final panel of 72 loci (see Table S1).

Global population level *F*_is_ calculated across all 72 loci varied between 0.006 (Wicklow—WK) and 0.22 (Cork Harbour—CKH) (details in Section 3.2).

### 3.2 Population Structure of Irish *Mytilus* spp.

**FIGURE 2:**
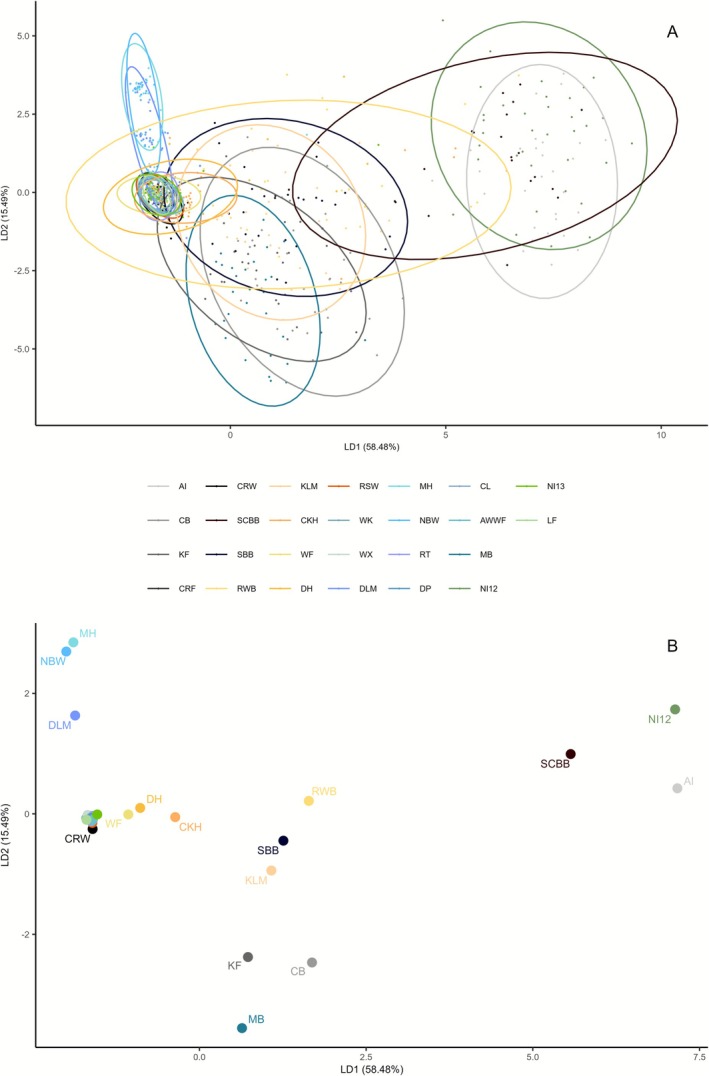
DAPC based on 72 SNP loci. (A) Individual‐level scatterplot, where each point represents a mussel individual. Colours indicate sampling sites, grouped by Irish coasts: Grey/black (west), yellow/orange (south), blue (east) and green (north, Republic of Ireland and Northern Ireland, UK). The percentage of variation explained by each discriminant function is shown on the axes. (B) Population‐level centroids from the same analysis. Each dot represents the average position of a sampling site in the discriminant space, labelled with its location code (see Table 1).

The DAPC was computed for all 26 Irish populations with the final panel of 72 loci (Figure 2 for DAPC), in which the first two eigenvalues accounted for 73.97% of the total variance, with linear discriminant 1 (LD1) accounting for almost 58.5% and LD2 for over 15%. The Irish populations clustered into four groups that overlapped to different degrees. The most distinct group separated along LD1 included two populations from the west of Ireland (Aran Islands—AI and Bertraghboy Bay—SCBB) and one from the North‐east (Dunseverick—NI12). Another distinct group consisted mainly of populations from the east coast but also included Cromane Farm (CRF) and Cromane Wild (CRW) from the southwest, which clustered with this group. Dún Laoghaire Marina (DLM), North Bull Wall (NBW) and Malahide (MH) on the east coast were slightly separated on the LD2 axis from the main east of Ireland cluster. The fourth group lay between the Aran Islands (AI), Bertraghboy Bay (SCBB) and Dunseverick (NI12) cluster and the east of Ireland and comprised mainly sites from the west and south coast of Ireland.

**FIGURE 3:**
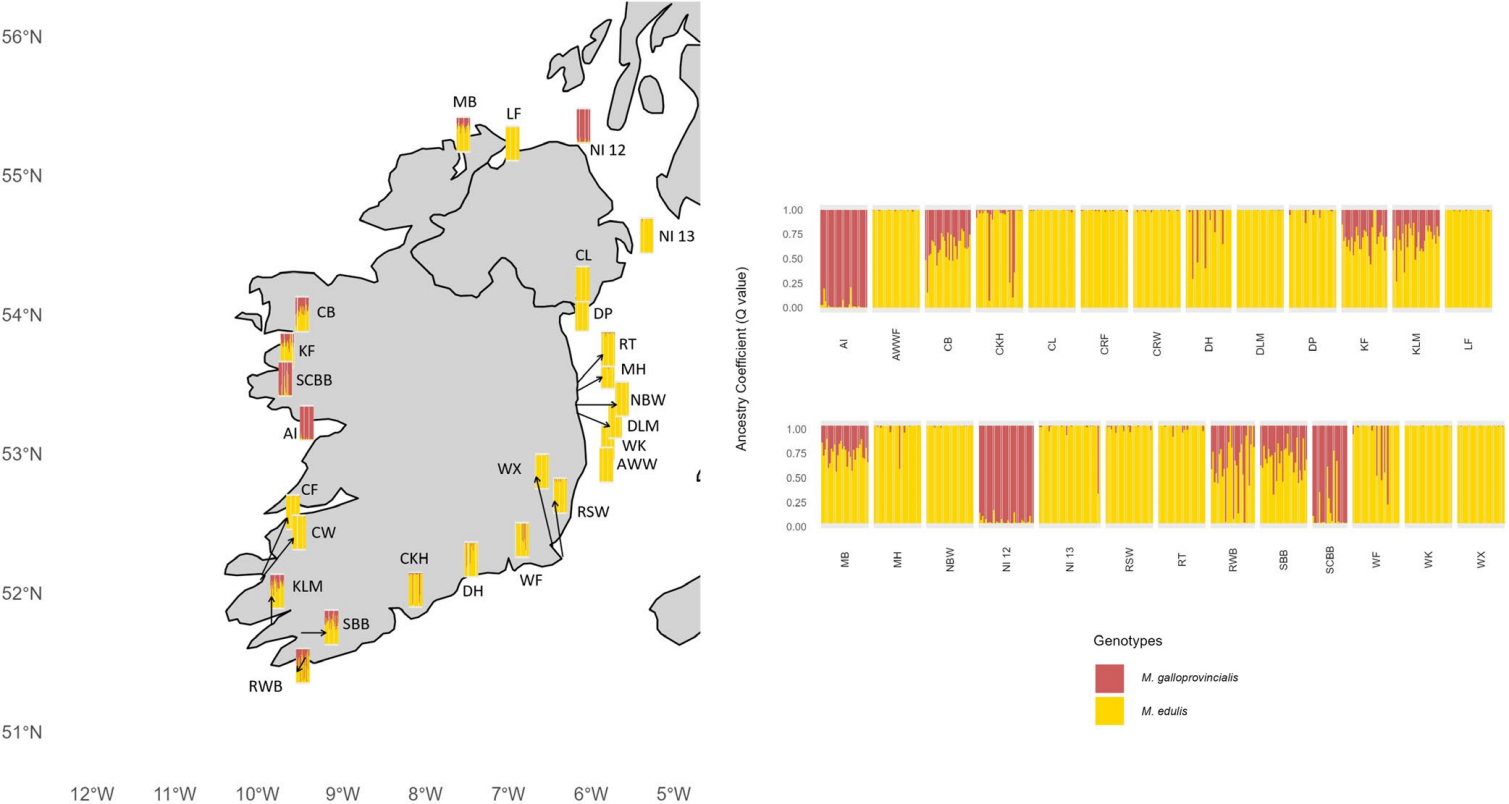
Results from STRUCTURE with best *K* = 2 to investigate the population structure, genetic admixture and ancestry inference of the Irish samples. Each column represents an individual, and individuals are grouped by populations on the *X* axis. The *Y* axis indicates the ancestry coefficient (*Q* value), and columns are coloured proportionally according to the composition of each of the two genotypes. Details on population codes are presented in Table 1.

The different grouping displayed by the DAPC showed a similar pattern in the STRUCTURE analysis, with the best *K* = 2 (Figure 3). The ancestry of individuals from Aran Islands (AI), Dunseverick (NI12) and Bertraghboy Bay (SCBB) was mainly composed of the *M. galloprovincialis* SNP genotypes, with an average *Q* value of 0.97 for both Aran Islands and Dunseverick, and 0.87 for Bertraghboy Bay. Populations from the east coast of Ireland, Cromane Farm and Cromane Wild showed a composition of mainly *M. edulis* SNP genotypes, with *Q* values > 0.9. Finally, samples from the west and south coast of Ireland showed a different proportion of both genotypes, with composition of *M. galloprovincialis* genotype ranging from 0.2 to 0.4. Therefore, four main groups can be identified from the DAPC and three groups with different ancestry composition can be identified from the STRUCTURE analyses: one group with a genetic make‐up of mainly *M. galloprovincialis* genotype, one group of exclusively *M. edulis* genotype and a third group that showed a composition of both genotypes, which indicates populations with different levels of admixture (from the west and south coast of Ireland—Clew Bay CB, Killary Fjord—KF, Kilmakilloge—KLM, Roaringwater Bay—RWB, Snave Bantry Bay—SBB, and one site from the north—Mulroy Bay MB).

**FIGURE 4:**
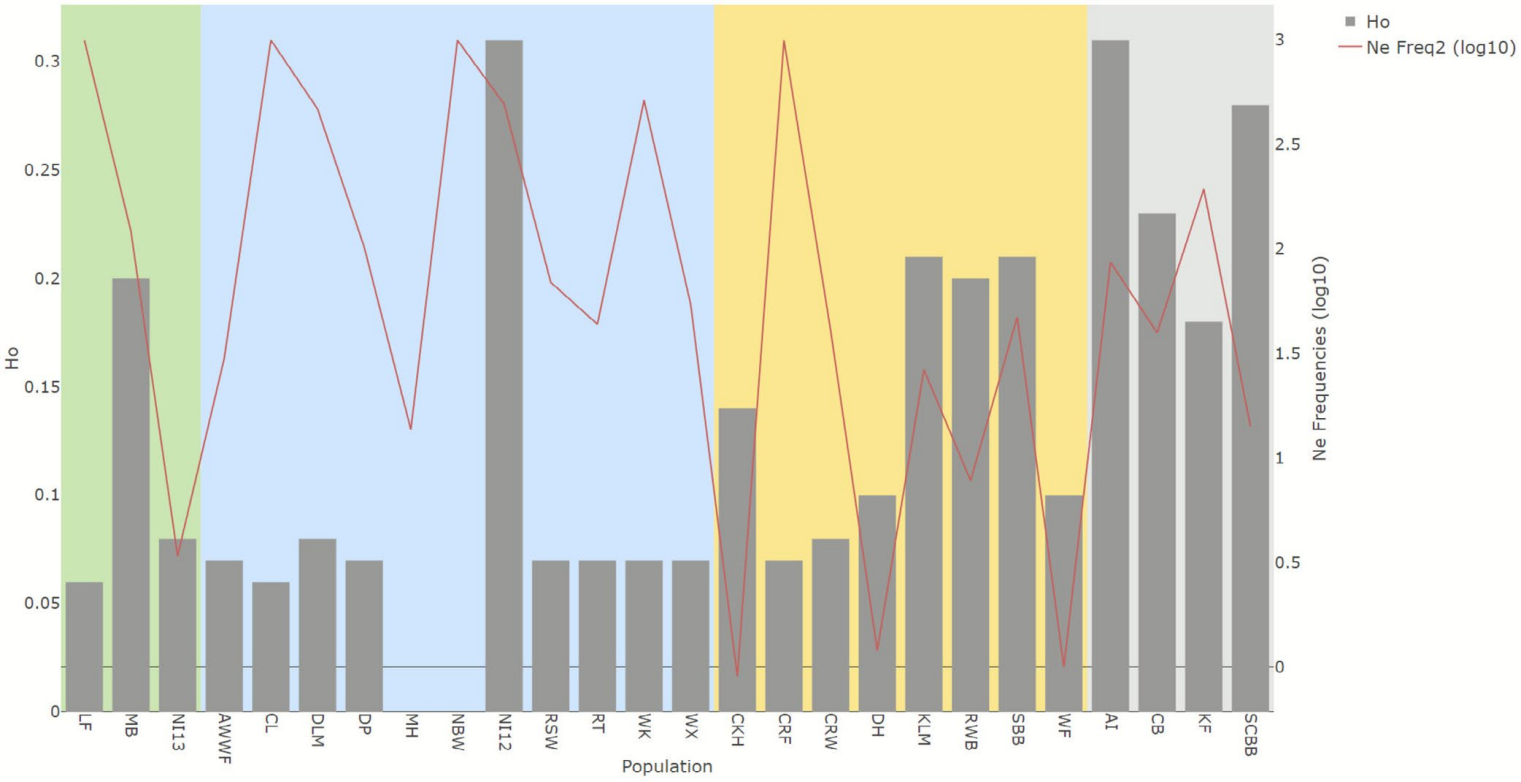
Observed heterozygosity (*H*_o_) (left *Y* axis, grey bars) and log_10_ transformed effective population size (*N*_e_) (right *Y* axis, red line) for the sampled Irish populations. *N*_e_ values that were ‘infinite’ were set at 1000 for the log_10_ transformation. *H*_o_ for Malahide and North Bull Wall was not possible to calculate. Grey shade background colour indicates sites from the west coast of Ireland, yellow background colour from the south coast, blue from the east coast and green from the north coast (Republic of Ireland and Northern Ireland, UK).

Allele richness (*A*_r_), observed heterozygosity (*H*_o_), expected heterozygosity (*H*_e_), and inbreeding coefficient (*F*_is_) calculated per population and averaged by genotype categories as indicated previously (Section 2.7), are reported in Table S2. Among populations, *A*_r_ ranged from a minimum of 1.28 (Dún Laoghaire Marina—DLM), to a maximum of 1.93 (Clew Bay—CB), *H*_o_ ranged from 0.06 (Lough Foyle—LF) to 0.31 (Aran Islands—AI and Dunseverick—NI12), *H*_e_ spanned from 0.07 (most of East coast populations and Cromane Farm—CRF) to 0.32 (Aran Islands, Dunseverick and Bertraghboy Bay—SCBB), and *F*_is_ from −0.02 (Dún Laoghaire Marina—DLM) to 0.22 (Cork Harbour—CKH). When looking at those indexes among the different genotypes, *H*_e_, *H*_o_, and *A*_r_ were higher in admixed populations and *M. galloprovincialis* (0.23–0.32, 0.20–0.3 and 1.85–1.886, respectively), while *M. edulis* populations showed substantially lower values (0.08, 0.075 and 1.39, respectively), which indicates much less genetic diversity. *F*_is_ varied between genotypes, with *M. edulis* having 0.08, *M. galloprovincialis* 0.11 and admixed populations 0.16. Effective population size (*N*_e_) calculated per population is reported in Table S3, and it ranged from a minimum of 0.9 (i.e., Cork Harbour) to infinite values (four populations; set at ‘1000’ to be log_10_ transformed). Ne was lowest (< 10) in Waterford Estuary (WF), Cork Harbour (CKH), Bangor (NI13), Dungarvan Harbour (DH) and Roaringwater Bay (RWB). The highest values (> 50) were found in Lough Foyle (LF), Carlingford Lough (CL), North Bull Wall (NBW), Aran Islands (AI), Cromane Farm (CRF), Killary Fjord (KF), Mulroy Bay (MB), Rosslare (RSW), Wicklow (WK), Wexford Harbour (WX), Dún Laoghaire Marina (DLM), Dunany Point (DP) and Dunseverick (NI12). Collectively, populations dominated by *M. edulis* genotype showed greater *N*_e_ compared to the *M. galloprovincialis* and mixed ancestry ones. When comparing populations for *H*_o_ and *N*_e_ (Figure 4), lower *H*_o_ and higher *N*_e_ values were observed in mostly east coast populations (Lough Foyle—LF, Carlingford Lough—CL, North Bull Wall—NBW, Dún Laoghaire Marina—DLM, Dunany Point—DP, Arklow Wind Farm—AWWF, Rogerstown—RT, Rosslare—RSW, Wexford Harbour—WX and Wicklow—WK) and Cromane sites (CRW and CRF), which suggests large populations with limited gene flow. Higher *H*_o_ and lower *N*_e_ were detected mainly in the west coast and south populations with consistent levels of admixture (i.e., Aran Islands—AI, Clew Bay—CB, Killary Fjord—KF, Bertraghboy Bay—SCBB, Kilmakilloge—KLM, Roaringwater Bay—RWB and Cork Harbour—CKH), which may indicate a recent admixture. Higher *H*_o_ coupled with higher *N*_e_ was detected in Dunseverick (NI12), potentially indicating large and genetically diverse populations. Finally, lower *H*_o_ coupled with lower *N*_e_, which suggests strong genetic drift or inbreeding was observed in Bangor (NI13), Dungarvan Harbour (DH) and Waterford Estuary (WF).

**FIGURE 5:**
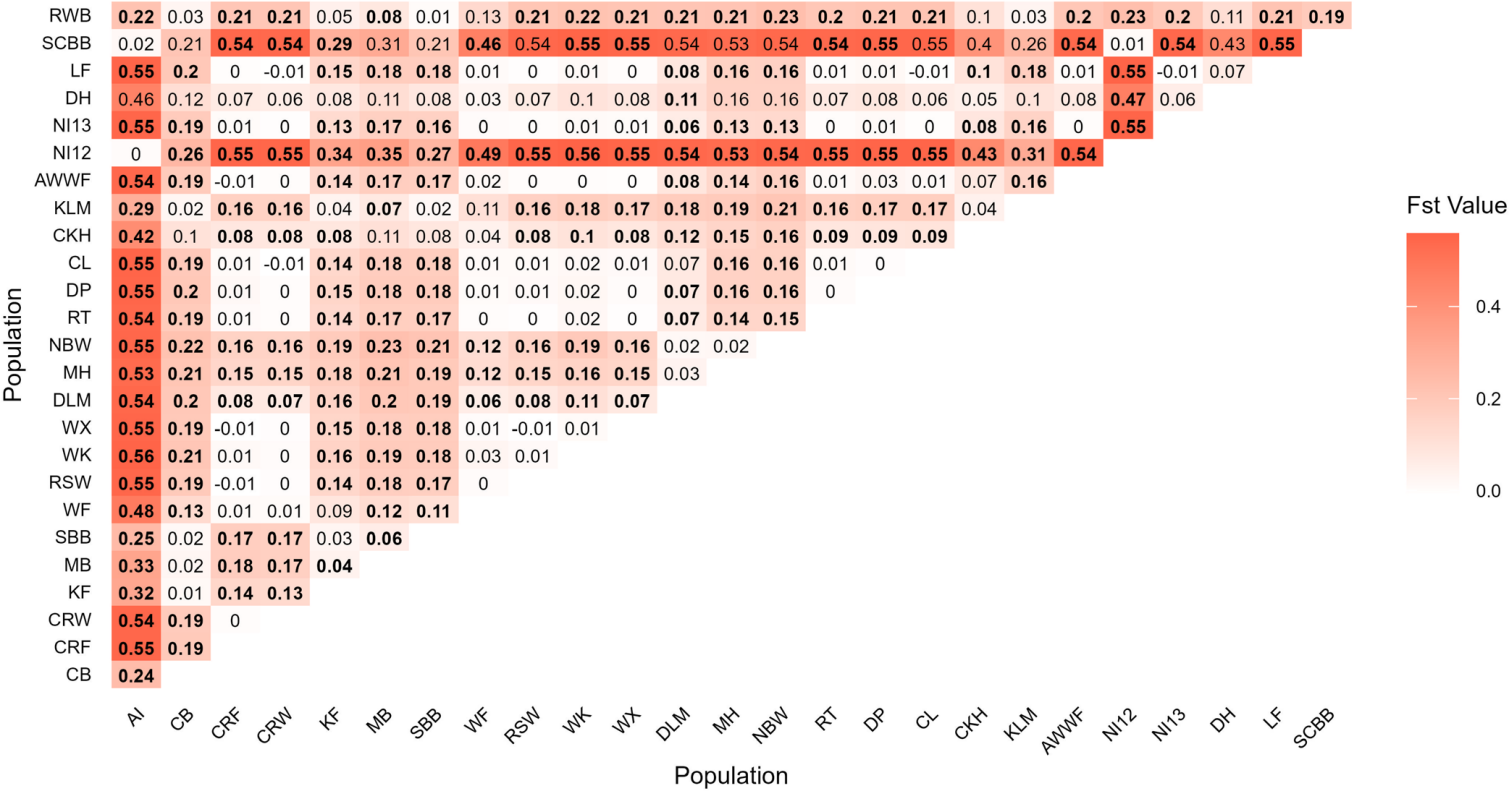
Heatmap of pairwise *F*_st_ with *p*‐values that are significant highlighted in bold. *F*_st_ and *p*‐values were calculated in *F*_stat_ where *p*‐values adjusted nominal level (5%) for multiple comparisons (Bonferroni) is 0.000154.

The pairwise *F*_st_ ranged from 0 to 0.56; the adjusted nominal level (5%) for multiple comparison with Bonferroni correction was set at 0.000154 (Figure 5). Overall, both the *F*_st_ values and the significance of the *p*‐value supported the separation of Irish mussels into four major groups. Aran Islands (AI) and Dunseverick (NI12) were significantly different from the rest of the locations, excluding Bertraghboy Bay (SCBB). Most of the locations on the east coast of Ireland were significantly different from the locations on the west coast (except for Cromane sites) and the Aran Islands, Dunseverick and Bertraghboy Bay. Finally, Malahide (MH), North Bull Wall (NBW) and Dún Laoghaire Marina (DLM) were found to be significantly different from most of the east coast locations, suggesting a substructure within this ‘pure’ Irish *M. edulis* genotype of the east coast.

### 3.3 *Mytilus edulis* Genotype Populations in Irish Mussels

**FIGURE 6:**
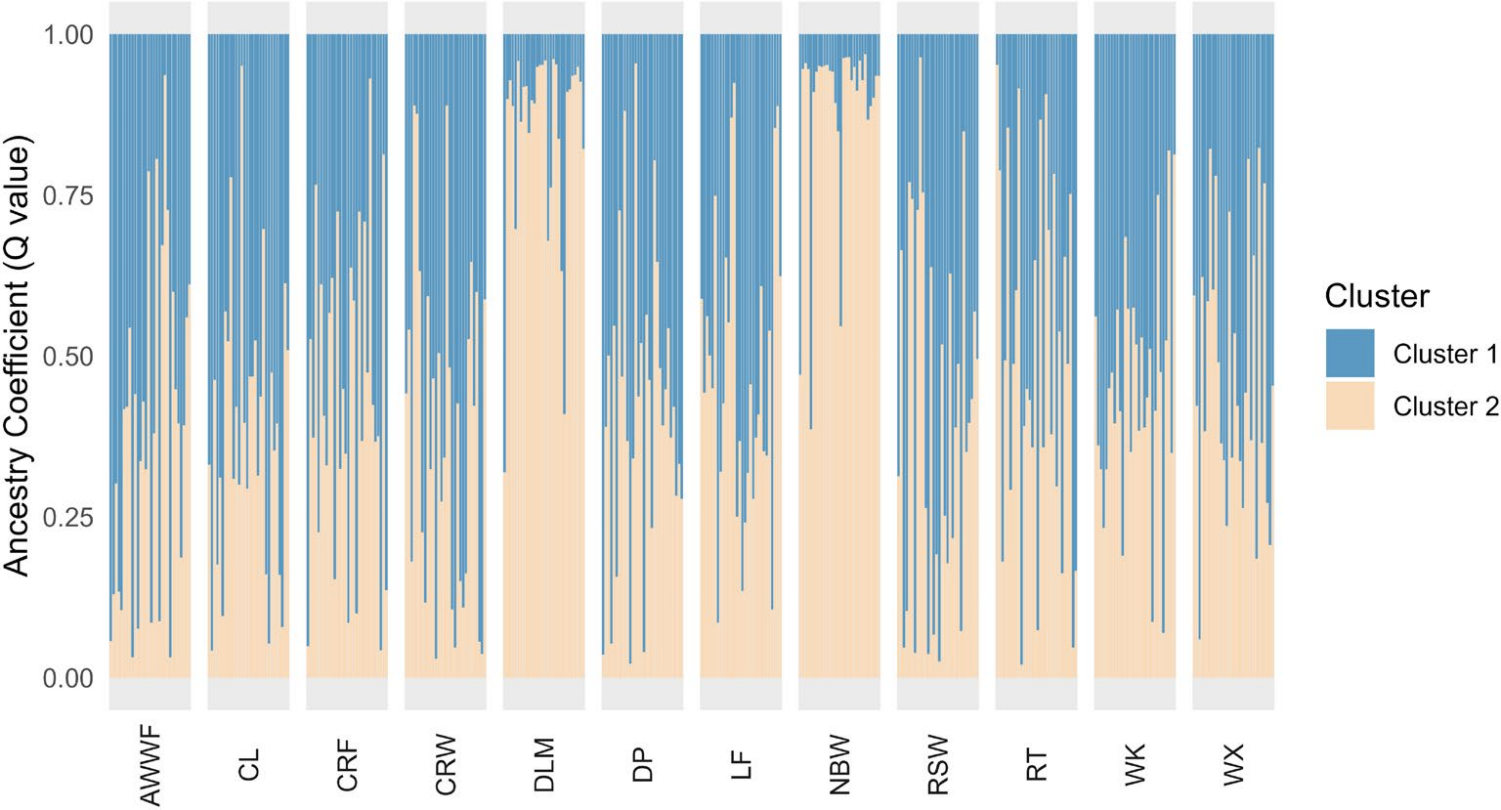
Results from STRUCTURE with best *K* = 2 to investigate the population structure of the ‘pure’ Irish *Mytilus edulis* genotype at a finer intraspecific level. Each column represents an individual, and individuals are grouped by populations on the *X* axis. The *Y* axis indicates the ancestry coefficient (*Q* value), and the columns are coloured proportionally according to the composition of each of the two intraspecific clusters.

The DAPC (Figure 2) and the pairwise *F*_st_ (Figure 5) analyses of Irish populations (Section 3.2) revealed further genetic structure among the Irish east coast samples of ‘pure’ *M. edulis* genotype (i.e., admixture proportion < 0.2). STRUCTURE analyses performed on two datasets (Figure S4 and Figure 6) identified the second dataset, which contained only ‘pure *M. edulis* ’ individuals, as the most appropriate to investigate the intraspecific population structure (12 populations). The DAPC (Figure S5) and STRUCTURE analyses (best *K* = 2; Figure 6) showed that mussels from Dún Laoghaire Marina (DLM) and North Bull Wall (NBW) grouped separately compared to the other populations, with an average ancestry coefficient for Cluster 1 of 0.1, while for the rest of the populations it ranged from 0.49 to 0.62.

### 3.4 Isolation by Distance and Correlation Between Genotype Composition, Pairwise *F*_st_ and Environmental Variables

**FIGURE 7:**
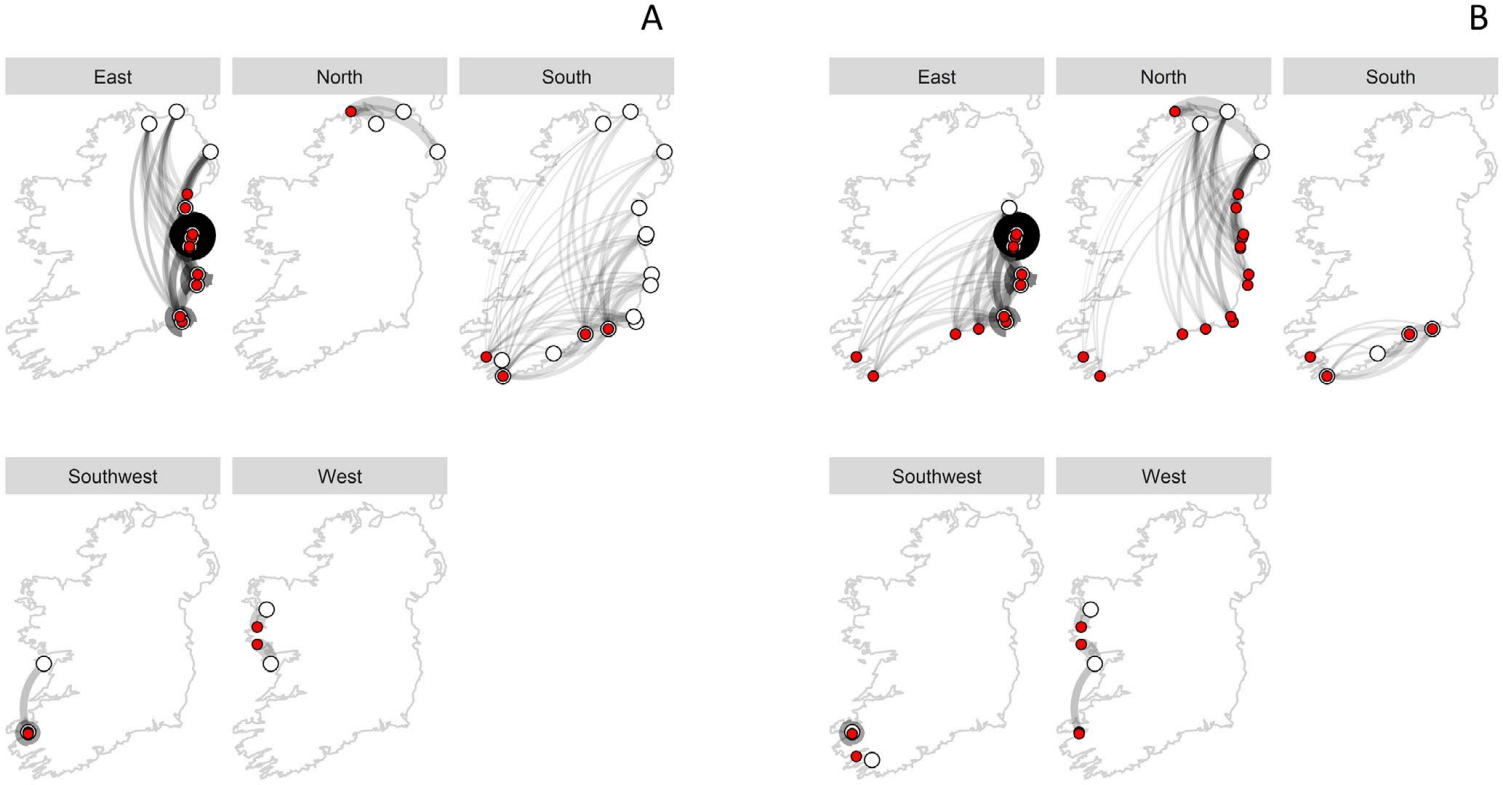
‘Sink‐source’ network map resulting from the IBD model showing the relative connectedness of the sites based on the mean current resistance between them. (A) map of connection to each region, (B) map of connection from each region. White dots are starting points (site a), red dots are end points (site b), and the thickness and transparency of the connection lines indicate the inverse of the seacost distance (i.e., the combination between currents and coastal distances): The thicker and darker the line, the better the connection is between site a and site b.

The results of the beta regression showed that pairwise genetic differentiation (*F*_st_) was significantly associated with ocean current resistance categories, as confirmed by the likelihood ratio test (LRT) (*χ* ^2^ = 14.91, df = 4, *p* = 0.0006; pseudo *R* ^2^ = 0.046) (Table 2). Post hoc tests confirmed that site pairs in the high‐resistance category (cat0) had higher *F*_st_ values (mean predicted *F*_st_ 0.197) than sites in the moderate current resistance category (cat1, mean predicted *F*_st_ 0.184), and site pairs in the low‐resistance category (cat2, mean predicted *F*_st_ 0.135). The only significant difference in *F*_st_ was detected between cat0 and cat2. These results are in line with the general assumption that population pairs with little connectivity via oceanographic pathways present a higher genetic differentiation. This pattern was observed in the IBD model (Figure 7), where the west and east coasts appeared to function as source regions, with east coast sites potentially receiving gene flow from the north, and the west coast sites contributing to the southwest, but not receiving from any other region. In contrast, the south region emerged as a sink, likely serving as the end point of connectivity from both the east and the southwest coast.

**TABLE 2:** Model results from beta regression analyses showing the top‐ranked models identified by the dredge function.

Model	Best fitting model	Pseudo‐*R*‐squared	Predictors	Model estimates (on logit scale)	*p*
Environmental	*Q*( *M. edulis* ) ~ min SST + max salinity + max wave height	0.24	Max salinity	−0.05	0.03*
			Max wave height	−0.89	< 0.001***
			Min SST	−0.14	0.12
IBD	*F*_st_ ~ connectivity	0.05	Cat0	−1.41	< 0.001***
			Cat1	−0.08	0.68
			Cat2	−0.45	< 0.001***
IBD + environmental	*F*_st_ ~ connectivity + Δwave height	0.14	Cat0	−1.94	< 0.001***
			Cat1	−0.10	0.60
			Cat2	−0.38	< 0.001***
			ΔWave Height	0.70	< 0.001***

*Note:* For each model, the pseudo‐*R* ^2^ is reported along with the parameter estimates (logit scale) and *p*‐values for predictors (* for significant *p*‐values). Response variables include ancestry coefficient for *Mytilus edulis* (*Q* values) and pairwise *F*_st_. Predictors include minimum sea surface temperature (min SST, in °C), maximum salinity (max salinity, in PSU), maximum significant wave height (max wave height, in meters), connectivity (categorical variable describing site‐to‐site connections in the IBD model), and absolute difference of maximum wave height between site pairs (Δwave height). Environmental variables were retrieved from the Regional Operational Model (ROMS) for the Northeast Atlantic (horizontal spatial resolution 1.9 km; temporal resolution of min SST and max salinity: 2017–2023; max wave height: 1994–2023). Significance codes: ***p < 0.001; **p < 0.01; *p < 0.05.

**FIGURE 8:**
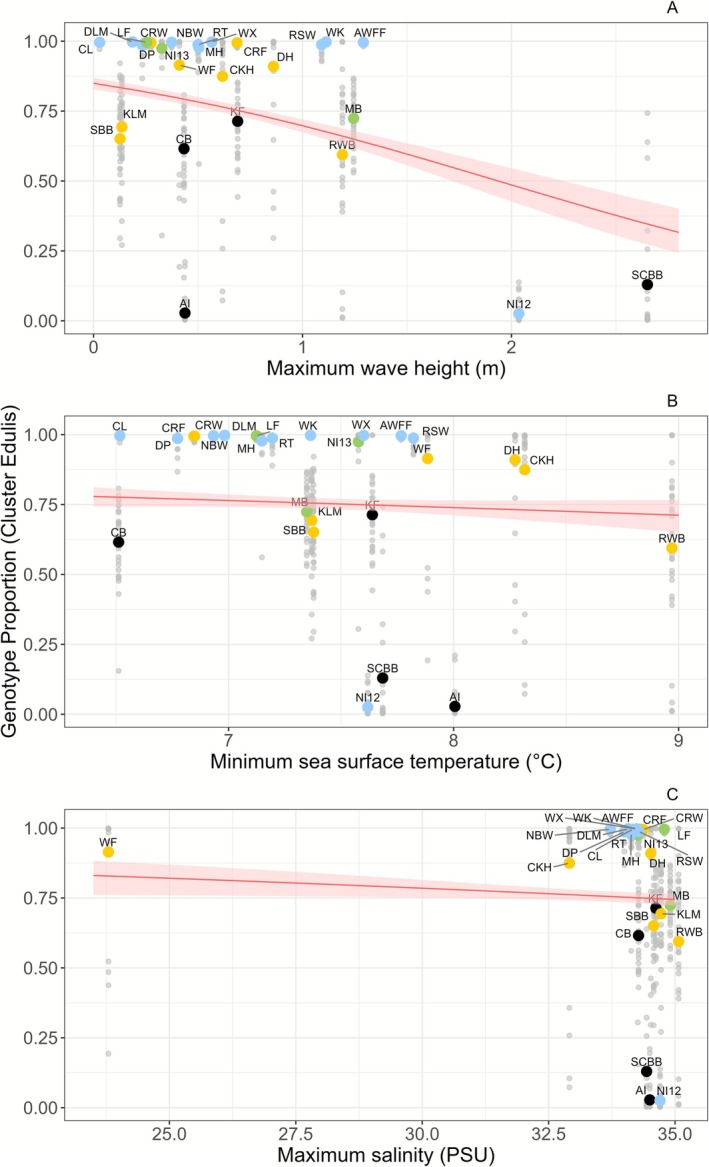
Scatterplots displaying the correlation between *Mytilus edulis* genotype proportion (*Q* values, *Y* axis) and environmental variables (*X* axis). (A) is maximum wave height (m), (B) is minimum sea surface temperature (°C), and (C) is maximum salinity (PSU). Red lines are beta regression predictors, with confidence interval shaded in pink. Grey dots are individuals' *Q* values, and coloured dots are the averaged populations' *Q* values. Colours indicate regions (blue east coast, yellow south coast, black west coast and green north coast), and details on population codes are presented in Table 1.

The results of the environmental factors beta regression showed that maximum wave height (max wave height), minimum sea surface temperature (min SST) and maximum salinity (max salinity) were the environmental variables that best explained the distribution of the *M. edulis* genotype across populations (Figure 8). All three variables together explained almost 24% of the total variability of genotype composition (pseudo‐*R* ^2^), and the LRT confirmed that the model including these predictors fit the data significantly better than the null model (*χ* ^2^ = 137.24, df = 3, *p* < 0.001) (Table 2). Maximum wave height was highly significant, maximum salinity was moderately significant, while minimum SST was not significant (all three showed negative effect sizes). These results collectively showed a negative correlation with *M. edulis* genotype composition, meaning that *M. edulis* genotype was more frequent at sheltered, cooler and fresher (i.e., lower salinity) sites. As shown in Figure 8, Waterford Estuary (WF) had much lower salinity than all other sites. Therefore, the leverage of the model, and an additional model was run without that site to check if max salinity was still a good predictor (Figure S7); the results corroborated the original model.

**FIGURE 9:**
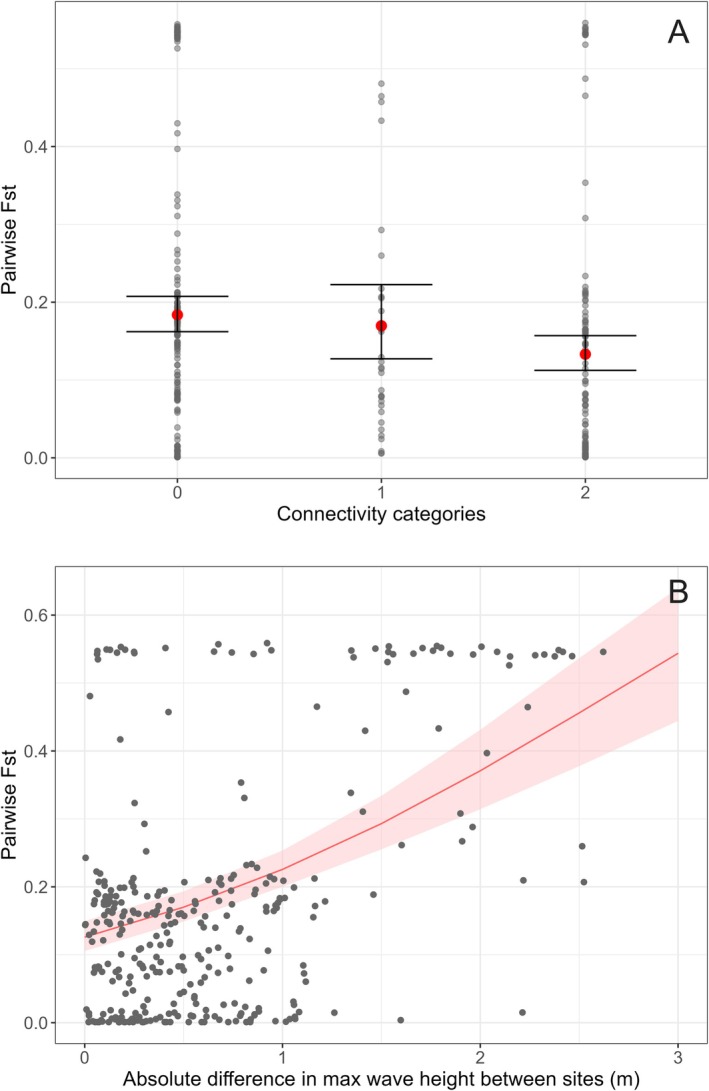
Scatterplots displaying the correlation between pairwise *F*_st_ (*Y* axis), and (A) connectivity category, and (B) absolute difference in maximum wave height between sites (Δ*m*). Connectivity category is the categorical variable describing site‐to‐site connections in the IBD model, where category 0 is unconnected, sites in category 1 have moderate connection and sites in category 2 have good connection. Red dots in (A) and red lines in (B) are model‐predicted marginal effects from the beta regression, with confidence interval shaded in pink. Grey dots are pairwise *F*_st_ values from the data.

The results of the final beta regression model (Figure 9) showed that between‐site genetic differentiation was significantly related to ocean current resistance and absolute difference in maximum wave height (inverse logit of the model estimate = 0.22, Table 2). The model explained 14% of the variation (*p* < 0.001; pseudo *R* ^2^ = 0.14). Post hoc tests confirmed that category 0 had the highest *F*_st_, followed by cat1, cat2. Overall, genetic differentiation was highest between sites unconnected by ocean currents and most distinct in terms of maximum wave height.

## 4 Discussion

### 4.1 Genetic Structure and Connectivity of the *Mytilus* Complex in the North‐East Atlantic: An Irish Case Study

This study provides the first SNP‐based population genomics investigation of the *Mytilus* species complex in Ireland, representing a key addition to regional efforts across the North‐east Atlantic. Because we used a set of multiple SNPs shared with other studies of hybrid zones in European waters (Hammel et al. 2021; Simon et al. 2018; Wilson et al. 2018) our results can be interpreted in the context of previously published patterns of *Mytilus* spp. genetic structure. Collectively, the results confirmed a distinct genetic structure among Irish coasts and highlighted how the use of a multi‐marker compared to a single‐marker approach can provide greater accuracy in resolving fine‐scale genetic structure, especially in admixed populations. In addition, this study explored how environmental and oceanographic features may shape genetic differentiation. While we refer to ‘connectivity’ throughout, we use this term to describe potential connectivity inferred from patterns of ocean current resistance and genetic differentiation.

Mussels from the east coast were composed almost exclusively of the *M. edulis* genotype, with evidence of intraspecific structuring in Dún Laoghaire Marina and North Bull Wall, and very limited admixture from *M. galloprovincialis* . In contrast, populations from the south and west coasts showed an increasing gradient of admixture: locations in the south‐east and south (i.e., Waterford Estuary, Cork Harbour and Dungarvan Harbour) presented few individuals with higher *Q* values of *M. galloprovincialis* genotype, but a higher and more consistent proportion of *M. galloprovincialis* ancestry was observed in populations in the south‐west and west. This pattern suggests that pure *M. edulis* populations dominate the Irish Sea, while more admixed populations are found along the southern and western coasts. This genetic structure is also supported by the pairwise *F*_st_ analysis and is in accordance with previous studies that used the single marker approach (i.e., Adhesive protein gene; Inoue et al. 1995) to show similar patterns that have remained relatively stable since the 1970s, with a clear distinction between the east coast of Ireland, dominated by *M. edulis* , and the *M. galloprovincialis* admixed populations prevailing in the south and west coasts (Gosling et al. 2008; Gosling and Wilkina 1981; Lynch et al. 2020). This distribution pattern that resembles a clinal gradient differs from the one observed in other *M. edulis* and *M. galloprovincialis* European hybrid zones: both in SW England and in the French Atlantic coast hybrid zones are characterised by a patchy composition often referred to as mosaic hybrid zones (Diz and Skibinski 2024; Fraïsse et al. 2016).

Beyond this broad clinal pattern, specific populations deviated from the expected structure gradient. The populations sampled in Cromane (southwest) were composed almost entirely of *M. edulis* despite their geographic location in a region where admixed populations are common. Similarly, populations from the Aran Islands (more specifically, Inis Meáin), Bertraghboy Bay (west coast) and Dunseverick (north coast) were dominated by *M. galloprovincialis* genotype individuals, in contrast to their neighbouring sites that showed a more admixed composition. These pockets of ‘pure’ *M. edulis* and *M. galloprovincialis* genotype populations within the admixture region along the west coast were not detected in previous studies (Coghlan and Gosling 2007; Gosling et al. 2008; Lynch et al. 2020). This discrepancy could be the result of patchy sampling coverage, the lack of overlap between the exact sites in this study and those in previous studies for these particular populations, or the higher resolution power of the SNP panel approach compared to the single marker method. As discussed by Larraín et al. (2019), multi‐locus approaches outperform single‐locus methods for characterising *Mytilus* spp. populations. In this study, a comparison between SNP panel genotyping and a single‐marker approach (adhesive protein gene for preliminary genotyping developed by Inoue et al. 1995, data available upon request) showed that while the single‐marker method is effective in populations with pure *M. edulis* ancestry (e.g., populations from the east coast), it produced significant discrepancies in populations with admixture, including those dominated by the *M. galloprovincialis* genotype. This highlights that while the single‐marker method is useful as a cost‐effective preliminary screening, this approach can misidentify species, especially for introgressed individuals. Thus, when resources are available, resolving genetic structure in admixed populations should be performed using a multi‐marker approach.

Oceanographic currents, their seasonal variability, and geographic distance are major drivers of genetic connectivity and structure in marine organisms with a pelagic larval stage (Coscia et al. 2020; Gilg and Hilbish 2003; Robins et al. 2013). Coastal currents around Ireland follow a broad clockwise direction from south to north‐west, and a gyre located in the Irish Sea often leads to self‐recruitment, that is, when the settlement of offspring is produced by the same population rather than by larvae dispersed from other populations (Brown et al. 2003; Emsley et al. 2005; Horsburgh and Hill 2003; Nolan et al. 2023; Sponaugle et al. 2014). The impact of the Irish Sea gyre on mussel population structure has been discussed by Gosling et al. (2008) and Robins et al. (2013), who hypothesised that the distinct *M. edulis* genotype population on the east coast could result from self‐recruitment due to the thermal fronts north and south of the Irish Sea. These fronts may act as soft barriers, limiting the influx of *Mytilus* spp. larvae, particularly during the spring spawning season.

In this study, the IBD model, which incorporates coastal distances weighted by oceanographic current resistance, revealed a clear pattern of relative isolation of the eastern and western sites, aligning with the Irish ocean currents system described above (Brown et al. 2003; Nolan et al. 2023). While the southern populations appeared well connected to both eastern and southwestern sites, the east coast primarily received input from the north, whereas west coast populations were largely isolated within the region, acting as sources only to south‐west locations. This pattern suggests potential local self‐recruitment driven by site‐specific hydrodynamic processes (Robins et al. 2013). This large‐scale pattern was significantly correlated with the pairwise *F*_st_ in the beta regression model, where greater distances and challenging dispersal paths corresponded to higher genetic differentiation. Similarly, a strong isolation by distance pattern was observed in the broadcast spawning species *Cerastoderma edule* in the Irish Sea (Coscia et al. 2020), supporting the idea that IBD is a primary driver of large‐scale genetic differentiation in broadcast spawners. However, at shorter distances and along lower resistance paths, the correlation between geographic distance and genetic differentiation was less pronounced. For example, the ‘Sink‐source’ network map (Figure 6) indicated potential connectivity between Cromane sites and the Aran Islands, a pattern not corroborated genetically. As reported by Demmer, Neill, et al. (2022), local oceanographic interactions, including wind‐driven and tidal dynamics, are essential for understanding finer‐scale larval dispersal and self‐recruitment in Irish mussel populations. Even in the absence of strong oceanographic barriers, site‐specific environmental conditions along with competition with local populations (i.e., *M. edulis* ) might prevent *M. galloprovincialis* from establishing in some areas, such as Cromane (Coscia et al. 2013; Knights et al. 2006). More in‐depth studies in these pure *M. edulis* and *M. galloprovincialis* sites among admixed regions could provide more insight into the processes that determine genetic composition.

The environmental drivers that shape the genomic composition of blue mussels, especially in the context of hybrid zone dynamics, have long been a focus of research, with fine‐scale environmental dynamics often playing a key role (Beaumont et al. 2004; Kijewski et al. 2019). In this study, environmental data for the spawning season (March–May) across 12 years identified maximum wave height and maximum salinity as the strongest predictors of *M. edulis* genotype composition. Wave exposure showed a significant negative association, followed by salinity, while sea surface temperature had a weaker, non‐significant effect. These results align with previous studies across European hybrid zones (including the Irish Atlantic coast), where the *M. galloprovincialis* genotype tends to dominate in wave‐exposed habitat, while *M. edulis* is more common in sheltered, freshwater‐influenced sites (Bierne, David, Langlade, and Bonhomme 2002; Gosling and Wilkina 1981). However, this pattern is not universally observed. For example, Hilbish et al. (2002) reported inconsistent spatial patterns in genotype distribution, and later studies in Ireland challenged the generality of these associations (Coghlan and Gosling 2007; Gosling et al. 2008).

The genotype‐environment association in *Mytilus* is more complex than previously assumed, likely influenced by local self‐recruitment patterns and the genetic signature of source adult populations. Coghlan and Gosling (2007) found no difference in the genetic composition between spat and adult populations, suggesting limited selective sorting at early life stages. Moreover, Lukić et al. (2024) reported no significant impact of wave exposure on *M. edulis* growth and survival under controlled experimental conditions. Additionally, other studies have linked fluctuations in *M. galloprovincialis* abundance along the Irish Atlantic coast to broader environmental shifts, such as rising sea surface temperatures (Gosling et al. 2008); however, colder spells and increased freshwater input seem to play a role in reducing the abundance of this genotype (Lynch et al. 2020), highlighting the role of episodic environmental variation. In this context, the results of this analysis reflect these observations: although wave height was the strongest predictor among the environmental variables investigated, other factors such as salinity and sea surface temperature might still influence shaping the local genomic composition of adult blue mussels.

Our model explained only 24% of the variation in genotype composition and was limited by a coarse resolution, especially in bays and fjords. As a result, the model reflects broader environmental patterns rather than capturing fine‐scale local dynamics. Moreover, other anthropogenic impacts can play a substantial role in the connectivity of mussel populations, shaping their genomic makeup (Simon et al. 2019). While the analysis of anthropogenic drivers (i.e., transplantation of mussel seed and spat, from wild seabed to farm bottom growth facilities; Bord Iascaigh Mhara 2024) is beyond the scope of this study, it remains an important avenue for future research.

To further explore how environmental factors interact with geographic isolation, we tested pairwise genetic differentiation (*F*_st_) in relation to both IBD and environmental distances. Our model showed that sea current resistance has the greater impact on population genetic differentiation in *Mytilus* spp. around the coast of Ireland, followed by wave height. This is consistent with what has been observed with cockle species in the Irish and Celtic Seas (Coscia et al. 2020). As discussed in Coscia et al. (2020), neutral genetic structure can strongly link with geographic and hydrodynamic dispersal potential, while environmental variables (such as SST) often are linked with non‐neutral loci, giving insight into potential drivers of adaptive divergence. In the current study, we did not test explicitly for adaptive divergence at our loci, but future work should strive to distinguish between adaptive vs. neutral loci, as the former class may exhibit stronger associations with environmental variables.

### 4.2 Genetic Diversity of Irish Blue Mussels

In this study, a clear pattern of genetic diversity was observed across Irish *Mytilus* spp. populations, enabled by the resolution provided by multi‐marker SNPs data. Despite being native to Irish coasts, *M. edulis* populations exhibit reduced genetic diversity but greater effective population sizes, while *M. galloprovincialis* and admixed populations maintain greater genetic diversity, likely shaped by gene flow and hybridisation. *M. galloprovincialis* genotype populations exhibited the highest levels of allelic richness (*A*_r_), observed heterozygosity (*H*_o_) and moderate effective population size (*N*_e_), followed by admixed populations, with *M. edulis* genotype populations showing the lowest values for *A*_r_ and *H*_o_. The effective population size resulting from this study is similar to the one observed by Gurney‐Smith et al. (2017), in which farmed mussel populations composed of mixed ancestry between *M. edulis* and *M. galloprovincialis* showed lower Ne compared to wild *M. edulis* . Moreover, the allelic richness and observed heterozygosity results are consistent with the variation in genetic diversity observed across *Mytilus* spp. populations by Vendrami et al. (2020), who also reported a higher genetic diversity in *M. galloprovincialis* genotype populations compared to *M. edulis* . Furthermore, they observed that introgression of *M. galloprovincialis* ancestry in *M. edulis* populations had a weak but positive effect on *H*_o_ level, suggesting that genetic diversity in admixed populations can be influenced by both the genetic background and the patterns and magnitude of introgression. The genetic diversity observed in Irish blue mussels aligns in part with that reported by Mathiesen et al. (2017). In both studies, admixed populations of *M. edulis* and *M. galloprovincialis* genotypes exhibited similar values of *A*_r_ and *H*_o_; however, a consistent difference emerged when comparing pure *M. edulis* and *M. galloprovincialis* genotype populations. Specifically, *M. edulis* populations in the Arctic region exhibited a higher diversity than the Irish *M. edulis* populations, while Irish *M. galloprovincialis* populations displayed higher genetic diversity compared to the reference Atlantic *M. galloprovincialis* included in the Mathiesen et al. (2017) study. These results suggest that Irish *M. galloprovincialis* and admixed populations maintain greater genetic diversity compared to pure Irish *M. edulis* populations, potentially due to differences in gene flow. The lower diversity in Irish *M. edulis* populations may reflect limited connectivity and high self‐recruitment as discussed in the previous section.

The inbreeding fixation index (*F*_is_), calculated across all loci for each population, was positive for most populations, with higher values in admixed and *M. galloprovincialis* genotype populations. The elevated *F*_is_ in admixed populations may reflect substructuring (Wahlund effect) if populations with different levels of introgression are pooled together (De Meeûs 2018). A similar pattern was observed in the southeast English hybrid zone, where Diz and Skibinski (2024) reported elevated *F*_is_ in hybrid populations. They suggested that multiple backcrossing and asymmetrical introgression between the parental genotypes could contribute to positive *F*_is_ values. To better understand the observed combination of higher genetic diversity and positive *F*_is_ in Irish admixed populations, future studies should aim to disentangle fine‐scale patterns of hybridisation and their relationship with *F*_is_. This could involve investigating individual ancestry proportions and *F*_is_, temporal variation in hybridisation dynamics and potential selection pressures acting on hybrids.

Collectively, these results indicate that despite being historically native to the Irish coast, Irish *M. edulis* populations may experience increased genetic isolation and relatively lower genetic diversity within the overall Irish context. In contrast, *M. galloprovincialis* and admixed populations exhibit higher genetic diversity and larger effective population sizes. As reported by Fraïsse et al. (2014, 2016) and Simon et al. (2018), hybridisation in *Mytilus* spp. populations could lead to a variety of introgression patterns (i.e., detrimental or beneficial effects), which play a central role in genetic diversity and adaptive potential. Genetic diversity and adaptive potential are key aspects in the aquaculture management framework, especially in the context of changing climate conditions (Brauer et al. 2023). As observed in *Crassostrea gigas* (Pacific oyster), crossbreeding between pure selected lines could increase genetic diversity and produce phenotypically superior offspring (Liang et al. 2023), at least in F1 hybrids owing to heterosis. However, these benefits may be short‐lived: in subsequent generations, recombination between divergent genomic backgrounds could potentially result in outbreeding depression and reduced fitness. Moreover, it is important to highlight that hybridisation in *Mytilus* spp. should be investigated on a case‐by‐case basis by employing larger panels of SNPs or Genome‐Wide approaches (e.g., RAD‐seq, WGS), as introgression could lead to unfavourable outcomes in certain hybrid zones, which may be detrimental for aquaculture (Dias et al. 2009; Dias, Piertney, et al. 2011; Gurney‐Smith et al. 2017; Nascimento‐Schulze et al. 2021).

### 4.3 Conclusion and Future Perspective

This study presents the first application of a 72 SNPs panel to investigate *Mytilus* spp. population genomics across 26 Irish populations, providing valuable genetic data for the North‐east Atlantic Ocean, and confirming a clear distinct genetic structure among the Irish coasts of previous studies. The multi‐marker approach offers a higher resolution of genetic admixture, diversity, isolation‐by‐distance and environmental associations. While regionally focused, these results contribute to broader knowledge on blue mussel hybridisation dynamics, population structure and environmental adaptation in temperate coastal ecosystems, and aquaculture management under climate change.

While this study provides a significant step towards better understanding the population structure of blue mussels in Irish waters, some limitations remain, including uneven geographic coverage and relatively small sample sizes. Thus, future work should address the following: (i) expand spatial sampling to monitor changes in *Mytilus* spp. population structure, especially in unsampled areas (e.g., Galway Bay, Sligo coast); (ii) targeted sampling of Irish *M. galloprovincialis* genotype populations, including key locations such as the Aran Islands, Bertraghboy Bay and Dunseverick; (iii) investigating genotype composition across different aquaculture practices; (iv) investigate fine‐scale local environmental‐genetic interactions by collecting high‐resolution environmental data; (v) including high‐resolution environmental and ocean circulation models to investigate connectivity and gene flow patterns; (vi) conducting genome‐wide association analyses to identify putative non‐neutral loci and hence better assess adaptive potential and (vii) investigating how different genotypes respond to environmental stressors to anticipate future responses to climate change.

## Funding

This work was supported by Bord Iascaigh Mhara (BIM) (RFT150321), European Commision, Marine Institute (Ireland), Department of Agriculture, Food and the Marine (DAFM, Ireland), Atlantic Technological University (Ireland).

## Conflicts of Interest

The authors declare no conflicts of interest.

## Supporting information


**Data S1:** eva70185‐sup‐0001‐supinfo.docx.

## Data Availability

The SNPs genotyping data that support the findings of this study are openly available in the Dryad Digital Repository at https://doi.org/10.5061/dryad.t1g1jwtgj.

## References

[eva70185-bib-0001] Avdelas, L. , E. Avdic‐Mravlje , A. C. Borges Marques , et al. 2021. “The Decline of Mussel Aquaculture in the European Union: Causes, Economic Impacts and Opportunities.” Reviews in Aquaculture 13, no. 1: 91–118. 10.1111/raq.12465.

[eva70185-bib-0002] Baden, S. , B. Hernroth , and O. Lindahl . 2021. “Declining Populations of *Mytilus* spp. in North Atlantic Coastal Waters‐A Swedish Perspective.” Journal of Shellfish Research 40, no. 2: 269–296. 10.2983/035.040.0207.

[eva70185-bib-0003] Barrett, N. J. , J. Thyrring , E. M. Harper , et al. 2022. “Molecular Responses to Thermal and Osmotic Stress in Arctic Intertidal Mussels ( *Mytilus edulis* ): The Limits of Resilience.” Genes 13, no. 1: 155. 10.3390/genes13010155. 35052494 PMC8774603

[eva70185-bib-0004] Bartoń, K. 2024. “MuMIn: Multi‐Model Inference.” R Package Version 1.48.4.

[eva70185-bib-0005] Beaumont, A. R. , M. P. Hawkins , F. L. Doig , I. M. Davies , and M. Snow . 2008. “Three Species of *Mytilus* and Their Hybrids Identified in a Scottish Loch: Natives, Relicts and Invaders?” Journal of Experimental Marine Biology and Ecology 367, no. 2: 100–110. 10.1016/j.jembe.2008.08.021.

[eva70185-bib-0006] Beaumont, A. R. , G. Turner , A. R. Wood , and D. O. F. Skibinski . 2004. “Hybridisations Between *Mytilus edulis* and *Mytilus galloprovincialis* and Performance of Pure Species and Hybrid Veliger Larvae at Different Temperatures.” Journal of Experimental Marine Biology and Ecology 302, no. 2: 177–188. 10.1016/j.jembe.2003.10.009.

[eva70185-bib-0007] Benabdelmouna, A. , and C. Ledu . 2016. “The Mass Mortality of Blue Mussels (*Mytilus* spp.) From the Atlantic Coast of France Is Associated With Heavy Genomic Abnormalities as Evidenced by Flow Cytometry.” Journal of Invertebrate Pathology 138: 30–38. 10.1016/j.jip.2016.06.001. 27264803

[eva70185-bib-0008] Bierne, N. , P. Borsa , C. Daguin , et al. 2003. “Introgression Patterns in the Mosaic Hybrid Zone Between *Mytilus edulis* and *M. galloprovincialis* .” Molecular Ecology 12, no. 2: 447–461. 10.1046/j.1365-294X.2003.01730.x. 12535095

[eva70185-bib-0009] Bierne, N. , P. David , P. Boudry , and F. Bonhomme . 2002. “Assortative Fertilization and Selection at Larval Stage in the Mussels *Mytilus edulis* and *M. galloprovincialis* .” Evolution 56, no. 2: 292–298. 10.1111/j.0014-3820.2002.tb01339.x. 11926497

[eva70185-bib-0010] Bierne, N. , P. David , A. Langlade , and F. Bonhomme . 2002. “Can Habitat Specialisation Maintain a Mosaic Hybrid.” Marine Ecology Progress Series 245: 157–170.

[eva70185-bib-0011] Bord Iascaigh Mhara . 2024. “BIM Annual Aquaculture Report 2024.” https://bim.ie/wp‐content/uploads/2024/10/BIM‐Aquaculture‐Report‐2024‐Final.pdf.

[eva70185-bib-0012] Braby, C. E. , and G. N. Somero . 2006. “Ecological Gradients and Relative Abundance of Native ( *Mytilus trossulus* ) and Invasive ( *Mytilus galloprovincialis* ) Blue Mussels in the California Hybrid Zone.” Marine Biology 148, no. 6: 1249–1262. 10.1007/s00227-005-0177-0.

[eva70185-bib-0013] Brauer, C. J. , J. Sandoval‐Castillo , K. Gates , et al. 2023. “Natural Hybridization Reduces Vulnerability to Climate Change.” Nature Climate Change 13, no. 3: 282–289. 10.1038/s41558-022-01585-1.

[eva70185-bib-0014] Brown, J. , L. Carrillo , L. Fernand , et al. 2003. “Observations of the Physical Structure and Seasonal Jet‐Like Circulation of the Celtic Sea and St. George's Channel of the Irish Sea.” Continental Shelf Research 23, no. 6: 533–561. 10.1016/S0278-4343(03)00008-6.

[eva70185-bib-0015] Brunson, J. C. , and Q. D. Read . 2023. “ggalluvial: Alluvial Plots in ‘ggplot2’.” R Package Version 0.12.5. http://corybrunson.github.io/ggalluvial/.

[eva70185-bib-0016] Canty, A. , and B. Ripley . 2022. “boot: Bootstrap R (S‐Plus) Functions.”

[eva70185-bib-0017] Coghlan, B. , and E. Gosling . 2007. “Genetic Structure of Hybrid Mussel Populations in the West of Ireland: Two Hypotheses Revisited.” Marine Biology 150, no. 5: 841–852. 10.1007/s00227-006-0408-z.

[eva70185-bib-0018] Cooney, R. , J. Dennis , E. Jackson , and C. Morrison . 2025. “Seafood Sustainability Progress Report: Aquaculture 2025.” https://bim.ie/wp‐content/uploads/2025/02/BIM‐Seafood‐Sustainability‐Progress‐Report‐Aquaculture.pdf.

[eva70185-bib-0019] Corrochano‐Fraile, A. , A. Davie , S. Carboni , and M. Bekaert . 2022. “Evidence of Multiple Genome Duplication Events in *Mytilus* Evolution.” BMC Genomics 23, no. 1: 340. 10.1186/s12864-022-08575-9. 35501689 PMC9063065

[eva70185-bib-0020] Coscia, I. , P. E. Robins , J. S. Porter , S. K. Malham , and J. E. Ironside . 2013. “Modelled Larval Dispersal and Measured Gene Flow: Seascape Genetics of the Common Cockle *Cerastoderma edule* in the Southern Irish Sea.” Conservation Genetics 14, no. 2: 451–466. 10.1007/s10592-012-0404-4.

[eva70185-bib-0021] Coscia, I. , S. B. Wilmes , J. E. Ironside , et al. 2020. “Fine‐Scale Seascape Genomics of an Exploited Marine Species, the Common Cockle *Cerastoderma edule*, Using a Multimodelling Approach.” Evolutionary Applications 13, no. 8: 1854–1867. 10.1111/eva.12932. 32908590 PMC7463313

[eva70185-bib-0022] Cribari‐Neto, F. , and A. Zeileis . 2010. “Beta Regression in R.” Journal of Statistical Software 34, no. 2: 1–24.

[eva70185-bib-0023] Davison, A. C. , and D. V. Hinkley . 1997. Bootstrap Methods and Their Applications. Cambridge University Press.

[eva70185-bib-0024] De Meeûs, T. 2018. “Revisiting F_IS_, F_ST_, Wahlund Effects, and Null Alleles.” Journal of Heredity 109, no. 4: 446–456. 10.1093/jhered/esx106. 29165594

[eva70185-bib-0025] del Rio‐Lavín, A. , N. Díaz‐Arce , M. A. Larraín , et al. 2022. “Population Structure and Geographic Origin Assignment of *Mytilus galloprovincialis* Mussels Using SNPs.” Aquaculture 550: 737836. 10.1016/j.aquaculture.2021.737836.

[eva70185-bib-0026] Demmer, J. , S. P. Neill , O. Andres , S. K. Malham , T. Jones , and P. Robins . 2022. “Larval Dispersal From an Energetic Tidal Channel and Implications for Blue Mussel ( *Mytilus edulis* ) Shellfisheries.” Aquaculture International 30, no. 6: 2969–2995. 10.1007/s10499-022-00948-x.

[eva70185-bib-0027] Demmer, J. , P. Robins , S. Malham , et al. 2022. “The Role of Wind in Controlling the Connectivity of Blue Mussels (*Mytilus edulis* L.) Populations.” Movement Ecology 10, no. 1: 3. 10.1186/s40462-022-00301-0. 35063034 PMC8783501

[eva70185-bib-0028] Dias, P. J. , M. Bland , A. M. Shanks , et al. 2009. “ *Mytilus* Species Under Rope Culture in Scotland: Implications for Management.” Aquaculture International 17, no. 5: 437–448. 10.1007/s10499-008-9214-6.

[eva70185-bib-0029] Dias, P. J. , B. Malgrange , M. Snow , and I. M. Davies . 2011. “Performance of Mussels, *Mytilus edulis*, *Mytilus trossulus*, and Their Hybrids in Cultivation at Three Scottish Lochs and Their Hybrids in Cultivation at Three Scottish Lochs.” Journal of the World Aquaculture Society 42, no. 1: 111–121.

[eva70185-bib-0030] Dias, P. J. , S. B. Piertney , M. Snow , and I. M. Davies . 2011. “Survey and Management of Mussel *Mytilus* Species in Scotland.” Hydrobiologia 670, no. 1: 127–140. 10.1007/s10750-011-0664-x.

[eva70185-bib-0031] Diz, A. P. , and D. O. F. Skibinski . 2024. “Patterns of Admixture and Introgression in a Mosaic *Mytilus galloprovincialis* and *Mytilus edulis* Hybrid Zone in SW England.” Molecular Ecology 33, no. 3: e17233. 10.1111/mec.17233. 38063472

[eva70185-bib-0032] Do, C. , R. S. Waples , D. Peel , G. M. Macbeth , B. J. Tillett , and J. R. Ovenden . 2014. “NeEstimator v2: Re‐Implementation of Software for the Estimation of Contemporary Effective Population Size (*N* _e_) From Genetic Data.” Molecular Ecology Resources 14, no. 1: 209–214. 10.1111/1755-0998.12157. 23992227

[eva70185-bib-0033] Doherty, S. D. , D. Brophy , and E. Gosling . 2009. “Synchronous Reproduction May Facilitate Introgression in a Hybrid Mussel (*Mytilus*) Population.” Journal of Experimental Marine Biology and Ecology 378, no. 1–2: 1–7. 10.1016/j.jembe.2009.04.022.

[eva70185-bib-0034] Emsley, S. M. , G. A. Tarling , and M. T. Burrows . 2005. “The Effect of Vertical Migration Strategy on Retention and Dispersion in the Irish Sea During Spring‐Summer.” Fisheries Oceanography 14, no. 3: 161–174. 10.1111/j.1365-2419.2005.00327.x.

[eva70185-bib-0035] Eurostat . n.d. “Aquaculture Statistics.” https://ec.europa.eu/eurostat/statistics‐explained/index.php?title=Aquaculture_statistics.

[eva70185-bib-0036] Evanno, G. , S. Regnaut , and J. Goudet . 2005. “Detecting the Number of Clusters of Individuals Using the Software STRUCTURE: A Simulation Study.” Molecular Ecology 14, no. 8: 2611–2620. 10.1111/j.1365-294X.2005.02553.x. 15969739

[eva70185-bib-0037] FAO . 2024. “The State of World Fisheries and Aquaculture 2024—Blue Transformation in Action.” In The State of World Fisheries and Aquaculture 2024. FAO. 10.4060/cd0683en.

[eva70185-bib-0038] Fly, E. K. , T. J. Hilbish , D. S. Wethey , and R. L. Rognstad . 2015. “Physiology and Biogeography: The Response of European Mussels (*Mytilus* spp.) to Climate Change.” American Malacological Bulletin 33, no. 1: 136–149. 10.4003/006.033.0111.

[eva70185-bib-0039] Fox, J. , and S. Weisberg . 2019. “An R Companion to Applied Regression.”

[eva70185-bib-0040] Fraïsse, C. , K. Belkhir , J. J. Welch , and N. Bierne . 2016. “Local Interspecies Introgression Is the Main Cause of Extreme Levels of Intraspecific Differentiation in Mussels.” Molecular Ecology 25, no. 1: 269–286. 10.1111/mec.13299. 26137909

[eva70185-bib-0041] Fraïsse, C. , C. Roux , J. J. Welch , and N. Bierne . 2014. “Gene‐Flow in a Mosaic Hybrid Zone: Is Local Introgression Adaptive?” Genetics 197, no. 3: 939–951. 10.1534/genetics.114.161380. 24788603 PMC4096372

[eva70185-bib-0042] Frantine, W. 2023. “ggDAPC: Visualize DAPC Results Using Ggplot [R Package].” GitHub. https://github.com/wilsonfrantine/ggDAPC.

[eva70185-bib-0043] Frichot, E. , and O. François . 2015. “LEA: An R Package for Landscape and Ecological Association Studies.” Methods in Ecology and Evolution 6, no. 8: 925–929. 10.1111/2041-210X.12382.

[eva70185-bib-0044] Gardner, J. P. A. , P. A. Oyarzún , J. E. Toro , R. Wenne , and M. Zbawicka . 2021. “Phylogeography of Southern Hemisphere Blue Mussels of the Genus *Mytilus*: Evolution, Biosecurity, Aquaculture and Food Labelling.” In Oceanography and Marine Biology: An Annual Review, vol. 59, 139–228. CRC Press. 10.1201/9781003138846-3.

[eva70185-bib-0045] Gardner, J. P. A. , and K. M. Westfall . 2012. “Geographic Distribution and Molecular Identification of a Metapopulation of Blue Mussels (Genus *Mytilus*) in Northeastern New Zealand.” Journal of Molluscan Studies 78, no. 1: 66–73. 10.1093/mollus/eyr037.

[eva70185-bib-0046] Gerdol, M. , R. Moreira , F. Cruz , et al. 2020. “Massive Gene Presence‐Absence Variation Shapes an Open Pan‐Genome in the Mediterranean Mussel.” Genome Biology 21, no. 1: 275. 10.1186/s13059-020-02180-3. 33168033 PMC7653742

[eva70185-bib-0047] Gilg, M. R. , and T. J. Hilbish . 2003. “Patterns of Larval Dispersal and Their Effect on the Maintenance of a Blue Mussel Hybrid Zone in Southwestern England.” Evolution 57, no. 5: 1061–1077. 10.1111/j.0014-3820.2003.tb00316.x. 12836823

[eva70185-bib-0048] Gosling, E. 2021. Marine Mussels: Ecology, Physiology, Genetics and Culture. John Wiley & Sons.

[eva70185-bib-0049] Gosling, E. , S. Doherty , and N. Howley . 2008. “Genetic Characterization of Hybrid Mussel (*Mytilus*) Populations on Irish Coasts.” Journal of the Marine Biological Association of the United Kingdom 88, no. 2: 341–346. 10.1017/S0025315408000957.

[eva70185-bib-0050] Gosling, E. , and N. Wilkina . 1981. “Ecological Genetics of the Mussels *Mytilus edulis* and *M. galloprovincialis* on Irish Coasts.” Marine Ecology Progress Series 4, no. 1975: 221–227. 10.3354/meps004221.

[eva70185-bib-0051] Goudet, J. 2001. “FSTAT, a Program to Estimate and Test Gene Diversities and Fixation Indices (Version 2.9.4). (2.9.4).”

[eva70185-bib-0052] Gruber, B. , P. J. Unmack , O. F. Berry , and A. Georges . 2018. “Dartr: An R Package to Facilitate Analysis of SNP Data Generated From Reduced Representation Genome Sequencing.” Molecular Ecology Resources 18, no. 3: 691–699. 10.1111/1755-0998.12745. 29266847

[eva70185-bib-0053] Gurney‐Smith, H. J. , A. J. Wade , and C. L. Abbott . 2017. “Species Composition and Genetic Diversity of Farmed Mussels in British Columbia, Canada.” Aquaculture 466: 33–40. 10.1016/j.aquaculture.2016.08.038.

[eva70185-bib-0054] Hammel, M. , A. Simon , C. Arbiol , et al. 2021. “Prevalence and Polymorphism of a Mussel Transmissible Cancer in Europe.” Molecular Ecology 31, no. 3: 736–751. 10.1111/mec.16052. 34192383 PMC8716645

[eva70185-bib-0055] Hijmans, R. 2025. “raster: Geographic Data Analysis and Modeling.”

[eva70185-bib-0056] Hilbish, T. J. , E. W. Carson , J. R. Plante , L. A. Weaver , and M. R. Gilg . 2002. “Distribution of *Mytilus edulis*, *M. galloprovincialis*, and Their Hybrids in Open‐Coast Populations of Mussels in Southwestern England.” Marine Biology 140, no. 1: 137–142. 10.1007/s002270100631.

[eva70185-bib-0057] Horsburgh, K. J. , and A. E. Hill . 2003. “A Three‐Dimensional Model of Density‐Driven Circulation in the Irish Sea.” Journal of Physical Oceanography 33: 343–365.

[eva70185-bib-0058] ICES . 2024. “Celtic Seas Ecoregion—Ecosystem Overviews in Report of the ICES Advisory Committee, 2024.” ICES Advice, 2024, Section 7.1. 10.17895/ices.advice.25713033.

[eva70185-bib-0059] Inoue, K. , J. H. Waite , M. Matsuoka , S. Odo , and S. Harayama . 1995. “Interspecific Variations in Adhesive Protein Sequences of *Mytilus edulis* , *M. galloprovincialis*, and *M. trossulus* .” Biological Bulletin 189, no. 3: 370–375. 10.2307/1542155. 8555320

[eva70185-bib-0060] Jombart, T. 2008. “Adegenet: A R Package for the Multivariate Analysis of Genetic Markers.” Bioinformatics 24, no. 11: 1403–1405. 10.1093/bioinformatics/btn129. 18397895

[eva70185-bib-0061] Jombart, T. , and I. Ahmed . 2011. “Adegenet 1.3‐1: New Tools for the Analysis of Genome‐Wide SNP Data.” Bioinformatics 27, no. 21: 3070–3071. 10.1093/bioinformatics/btr521. 21926124 PMC3198581

[eva70185-bib-0062] Keenan, K. , P. Mcginnity , T. F. Cross , W. W. Crozier , and P. A. Prodöhl . 2013. “DiveRsity: An R Package for the Estimation and Exploration of Population Genetics Parameters and Their Associated Errors.” Methods in Ecology and Evolution 4, no. 8: 782–788. 10.1111/2041-210X.12067.

[eva70185-bib-0063] Kenchington, E. L. , B. W. MacDonald , A. Cogswell , L. C. Hamilton , and A. P. Diz . 2020. “Sex‐Specific Effects of Hybridization on Reproductive Fitness in *Mytilus* .” Journal of Zoological Systematics and Evolutionary Research 58, no. 2: 581–597. 10.1111/jzs.12348.

[eva70185-bib-0064] Kijewski, T. , B. Śmietanka , M. Zbawicka , E. Gosling , H. Hummel , and R. Wenne . 2011. “Distribution of *Mytilus* Taxa in European Coastal Areas as Inferred From Molecular Markers.” Journal of Sea Research 65, no. 2: 224–234. 10.1016/j.seares.2010.10.004.

[eva70185-bib-0065] Kijewski, T. , M. Zbawicka , J. Strand , et al. 2019. “Random Forest Assessment of Correlation Between Environmental Factors and Genetic Differentiation of Populations: Case of Marine Mussels *Mytilus* .” Oceanologia 61, no. 1: 131–142. 10.1016/j.oceano.2018.08.002.

[eva70185-bib-0066] Knights, A. M. , T. P. Crowe , and G. Burnell . 2006. “Mechanisms of Larval Transport: Vertical Distribution of Bivalve Larvae Varies With Tidal Conditions.” Marine Ecology Progress Series 326: 167–174.

[eva70185-bib-0067] Kopelman, N. M. , J. Mayzel , M. Jakobsson , N. A. Rosenberg , and I. Mayrose . 2015. “Clumpak: A Program for Identifying Clustering Modes and Packaging Population Structure Inferences Across K.” Molecular Ecology Resources 15, no. 5: 1179–1191. 10.1111/1755-0998.12387. 25684545 PMC4534335

[eva70185-bib-0068] Larraín, M. A. , P. González , C. Pérez , and C. Araneda . 2019. “Comparison Between Single and Multi‐Locus Approaches for Specimen Identification in *Mytilus* Mussels.” Scientific Reports 9, no. 1: 19714. 10.1038/s41598-019-55855-8. 31873129 PMC6928075

[eva70185-bib-0069] Larraín, M. A. , M. Zbawicka , C. Araneda , J. P. A. Gardner , and R. Wenne . 2018. “Native and Invasive Taxa on the Pacific Coast of South America: Impacts on Aquaculture, Traceability and Biodiversity of Blue Mussels (*Mytilus* spp.).” Evolutionary Applications 11, no. 3: 298–311. 10.1111/eva.12553.

[eva70185-bib-0070] Lenth, R. 2025. “emmeans: Estimated Marginal Means, Aka Least‐Squares Means.”

[eva70185-bib-0071] Liang, Y. , C. Xu , and Q. Li . 2023. “Heterosis and Genetic Diversity of Intraspecific Hybrids Crosses Between Two Selected Lines of the Pacific Oyster *Crassostrea gigas* .” Aquaculture 569: 739369. 10.1016/j.aquaculture.2023.739369.

[eva70185-bib-0072] Little, C. , C. D. Trowbridge , M. Pilling , G. A. Williams , D. Morritt , and P. Stirling . 2024. “Long‐Term Fluctuations and Recent Decline of Mussel Populations in an Irish Sea Lough.” Journal of Molluscan Studies 90, no. 1: eyae002. 10.1093/mollus/eyae002.

[eva70185-bib-0073] Lüdecke, D. 2018. “Ggeffects: Tidy Data Frames of Marginal Effects From Regression Models.” Journal of Open Source Software 3, no. 26: 772. 10.21105/joss.00772.

[eva70185-bib-0074] Lukić, I. , L. Hayes , and T. Bekkby . 2024. “Low to Moderate Wave Exposure Did Not Impact Blue Mussel (*Mytilus edulis*) Growth in a Mesocosm Study.” PLoS One 19, no. 12: e0315136. 10.1371/journal.pone.0315136. 39637144 PMC11620627

[eva70185-bib-0075] Lupo, C. , S. Bougeard , V. Le Bihan , et al. 2021. “Mortality of Marine Mussels *Mytilus edulis* and *M. galloprovincialis*: Systematic Literature Review of Risk Factors and Recommendations for Future Research.” Reviews in Aquaculture 13, no. 1: 504–536. 10.1111/raq.12484.

[eva70185-bib-0076] Lynch, S. A. , A. Coghlan , B. O. Leary , E. Morgan , and S. C. Culloty . 2020. “Northward Establishment of the Mediterranean Mussel *Mytilus galloprovincialis* Limited by Changing Climate.” Biological Invasions 22, no. 9: 2725–2736. 10.1007/s10530-020-02294-6.

[eva70185-bib-0077] Massicotte, P. , and A. South . 2023. “rnaturalearth: World Map Data From Natural Earth.”

[eva70185-bib-0078] Mathiesen, S. S. , J. Thyrring , J. Hemmer‐Hansen , et al. 2017. “Genetic Diversity and Connectivity Within *Mytilus* spp. in the Subarctic and Arctic.” Evolutionary Applications 10, no. 1: 39–55. 10.1111/eva.12415. 28035234 PMC5192891

[eva70185-bib-0079] McDevitt, A. D. , I. Coscia , S. S. Browett , et al. 2022. “Next‐Generation Phylogeography Resolves Post‐Glacial Colonization Patterns in a Widespread Carnivore, the Red Fox (*Vulpes vulpes*), in Europe.” Molecular Ecology 31, no. 3: 993–1006. 10.1111/mec.16276. 34775636

[eva70185-bib-0080] Michalek, K. , A. Ventura , and T. Sanders . 2016. “ *Mytilus* Hybridisation and Impact on Aquaculture: A Minireview.” Marine Genomics 27: 3–7. 10.1016/j.margen.2016.04.008. 27157133

[eva70185-bib-0081] Mijangos, J. L. , B. Gruber , O. Berry , C. Pacioni , and A. Georges . 2022. “dartR v2: An Accessible Genetic Analysis Platform for Conservation, Ecology and Agriculture.” Methods in Ecology and Evolution 13, no. 10: 2150–2158. 10.1111/2041-210X.13918.

[eva70185-bib-0082] Murgarella, M. , D. Puiu , B. Novoa , A. Figueras , D. Posada , and C. Canchaya . 2016. “A First Insight Into the Genome of the Filter‐Feeder Mussel *Mytilus galloprovincialis* .” PLoS One 11, no. 3: e0151561. 10.1371/journal.pone.0151561. 26977809 PMC4792442

[eva70185-bib-0083] Nagy, H. , K. Lyons , G. Nolan , M. Cure , and T. Dabrowski . 2020. “A Regional Operational Model for the North East Atlantic: Model Configuration and Validation.” Journal of Marine Science and Engineering 8, no. 9: 1–27. 10.3390/jmse8090673.

[eva70185-bib-0084] Nascimento‐Schulze, J. C. , T. P. Bean , R. D. Houston , et al. 2021. “Optimizing Hatchery Practices for Genetic Improvement of Marine Bivalves.” Reviews in Aquaculture 13, no. 4: 2289–2304. 10.1111/raq.12568.

[eva70185-bib-0085] Nascimento‐Schulze, J. C. , T. P. Bean , C. Peñaloza , et al. 2023. “SNP Discovery and Genetic Structure in Blue Mussel Species Using Low Coverage Sequencing and a Medium Density 60 K SNP‐Array.” Evolutionary Applications 16, no. 5: 1044–1060. 10.1111/eva.13552. 37216031 PMC10197230

[eva70185-bib-0086] Nolan, G. , C. Cusack , and D. Fitzhenry , eds. 2023. Irish Ocean Climate & Ecosystem Status Report. Marine Institute.

[eva70185-bib-0087] Pritchard, J. K. , M. Stephens , and P. Donnelly . 2000. “Inference of Population Structure Using Multilocus Genotype Data.” http://www.stats.ox.ac.uk/pritch/home.html. 10.1093/genetics/155.2.945 PMC1461096 10835412

[eva70185-bib-0088] R Core Team . 2023. R: A Language and Environment for Statistical Computing. R Foundation for Statistical Computing.

[eva70185-bib-0089] Regan, T. , T. S. Hori , and T. P. Bean . 2024. “A Chromosome‐Scale *Mytilus edulis* Genome Assembly for Aquaculture, Marine Ecology, and Evolution.” G3: Genes, Genomes, Genetics 14, no. 8: jkae138. 10.1093/g3journal/jkae138. 38935082 PMC11304980

[eva70185-bib-0090] Robins, P. E. , S. P. Neill , L. Giménez , R. Stuart , S. R. Jenkins , and S. K. Malham . 2013. “Physical and Biological Controls on Larval Dispersal and Connectivity in a Highly Energetic Shelf Sea.” Limnology and Oceanography 58, no. 2: 505–524. 10.4319/lo.2013.58.2.0505.

[eva70185-bib-0091] Robinson, Z. 2024. “RLDNe: A Convenient R Interface for NeEstimator V2.1.”

[eva70185-bib-0092] Rousset, F. 2008. “GENEPOP'007: A Complete Re‐Implementation of the GENEPOP Software for Windows and Linux.” Molecular Ecology Resources 8, no. 1: 103–106. 10.1111/j.1471-8286.2007.01931.x. 21585727

[eva70185-bib-0093] Seed, R. 1974. “Morphological Variations in *Mytilus* From the Irish Coasts in Relation to the Occurrence and Distribution of *M. galloprovincialis* Lmk.”

[eva70185-bib-0094] Shields, J. L. , P. Barnes , and D. D. Heath . 2008. “Growth and Survival Differences Among Native, Introduced and Hybrid Blue Mussels (*Mytilus* spp.): Genotype, Environment and Interaction Effects.” Marine Biology 154, no. 5: 919–928. 10.1007/s00227-008-0985-0.

[eva70185-bib-0095] Simon, A. , C. Arbiol , E. E. Nielsen , et al. 2019. “Replicated Anthropogenic Hybridisations Reveal Parallel Patterns of Admixture in Marine Mussels.” Evolutionary Applications 13: 575–599. 10.1111/eva.12879. 32431737 PMC7045717

[eva70185-bib-0096] Simon, A. , N. Bierne , and J. J. Welch . 2018. “Coadapted Genomes and Selection on Hybrids: Fisher's Geometric Model Explains a Variety of Empirical Patterns.” Evolution Letters 2, no. 5: 472–498. 10.1002/evl3.66. 30283696 PMC6145440

[eva70185-bib-0097] Simon, A. , C. Fraïsse , T. El Ayari , et al. 2021. “How Do Species Barriers Decay? Concordance and Local Introgression in Mosaic Hybrid Zones of Mussels.” Journal of Evolutionary Biology 34, no. 1: 208–223. 10.1111/jeb.13709. 33045123

[eva70185-bib-0098] Sponaugle, S. , A. L. Shanks , S. Gaines Morgan , and J. M. Leis . 2014. “Predicting Self‐Recruitment in Marine Populations: Biophysical Correlates and Mechanisms.” https://www.researchgate.net/publication/233568480.

[eva70185-bib-0099] Standard BioTools . n.d. “Standard Bio Tools SNP Genotyping Analysis Software v.1.0.2.” https://www.standardbio.com/.

[eva70185-bib-0100] Stuckas, H. , L. Knöbel , H. Schade , et al. 2017. “Combining Hydrodynamic Modelling With Genetics: Can Passive Larval Drift Shape the Genetic Structure of Baltic *Mytilus* Populations?” Molecular Ecology 26, no. 10: 2765–2782. 10.1111/mec.14075. 28238204

[eva70185-bib-0101] Väinölä, R. , and P. Strelkov . 2011. “ *Mytilus trossulus* in Northern Europe.” Marine Biology 158, no. 4: 817–833. 10.1007/s00227-010-1609-z. 24391261 PMC3873017

[eva70185-bib-0102] van der Schatte Olivier, A. , L. Jones , L. L. Vay , M. Christie , J. Wilson , and S. K. Malham . 2020. “A Global Review of the Ecosystem Services Provided by Bivalve Aquaculture.” Reviews in Aquaculture 12, no. 1: 3–25. 10.1111/raq.12301.

[eva70185-bib-0103] van Etten, J. 2017. “R Package gdistance: Distances and Routes on Geographical Grids.” Journal of Statistical Software 76, no. 1: 1–21. 10.18637/jss.v076.i13.36568334

[eva70185-bib-0104] Vendrami, D. L. J. , M. De Noia , L. Telesca , E. M. Brodte , and J. I. Hoffman . 2020. “Genome‐Wide Insights Into Introgression and Its Consequences for Genome‐Wide Heterozygosity in the *Mytilus* Species Complex Across Europe.” Evolutionary Applications 13, no. 8: 2130–2142. 10.1111/eva.12974. 32908609 PMC7463347

[eva70185-bib-0105] Wenne, R. , A. Prądzińska , A. Poćwierz‐Kotus , M. A. Larraín , C. Araneda , and M. Zbawicka . 2022. “Provenance of *Mytilus* Food Products in Europe Using SNP Genetic Markers.” Aquaculture 554: 738135. 10.1016/j.aquaculture.2022.738135.

[eva70185-bib-0106] Wenne, R. , M. Zbawicka , L. Bach , et al. 2020. “Trans‐Atlantic Distribution and Introgression as Inferred From Single Nucleotide Polymorphism: Mussels *Mytilus* and Environmental Factors.” Genes 11, no. 5: 530. 10.3390/genes11050530. 32397617 PMC7288462

[eva70185-bib-0107] Wickham, H. , W. Chang , L. Henry , et al. 2016. ggplot2: Elegant Graphics for Data Analysis. Springer‐Verlag.

[eva70185-bib-0108] Wilson, J. , I. Matejusova , R. E. McIntosh , S. Carboni , and M. Bekaert . 2018. “New Diagnostic SNP Molecular Markers for the *Mytilus* Species Complex.” PLoS One 13, no. 7: e0200654. 10.1371/journal.pone.0200654. 30001394 PMC6042762

[eva70185-bib-0109] Yaghubi, E. , S. Carboni , R. M. J. Snipe , et al. 2021. “Farmed Mussels: A Nutritive Protein Source, Rich in Omega‐3 Fatty Acids, With a Low Environmental Footprint.” Nutrients 13, no. 4: 1124. 10.3390/nu13041124. 33805534 PMC8067026

[eva70185-bib-0110] Zbawicka, M. , R. Wenne , P. J. Dias , and J. P. A. Gardner . 2022. “Combined Threats to Native Smooth‐Shelled Mussels (Genus *Mytilus*) in Australia: Bioinvasions and Hybridization.” Zoological Journal of the Linnean Society 194: 1194–1211.

