# Supplementary material for: Population Genomics and Connectivity of the Blue Mussel Species Complex: Insights From a North‐East Atlantic Hybrid Zone
Source: Evol Appl. 2025 Nov 27;18(12):e70185. doi: 10.1111/eva.70185 (PMC12658909; doi:10.1111/eva.70185)
Supplement: Supplementary file 1 — Data S1: eva70185‐sup‐0001‐supinfo.docx. [file EVA-18-e70185-s001.docx]

**Supplemental Information for:**

**Population genomics and connectivity of the blue mussel species complex: insights from a North-east Atlantic hybrid zone**

**Table of Contents:**

| Figure S1 | Page 2 |
| --- | --- |
| Figure S2 | Page 3 |
| Figure S3 | Page 4 |
| Figure S4 | Page 5 |
| Figure S5 | Page 6 |
| Figure S6 | Page 7 |
| Figure S7 | Page 8 |
| Table S1 | Page 9 |
| Table S2 | Page 12 |
| Table S3 | Page 13 |
| Table S4 | Page 14 |
| Reference Genomes details | Page 15 |
| Supplementary Methods S1: Protocol optimization for SNPs genotyping on microfluidic Biomark HD platform | Page 16 |
| Supplementary Methods S2: Sea Current Resistance Modelling | Page 17 |
| References | Page 19 |


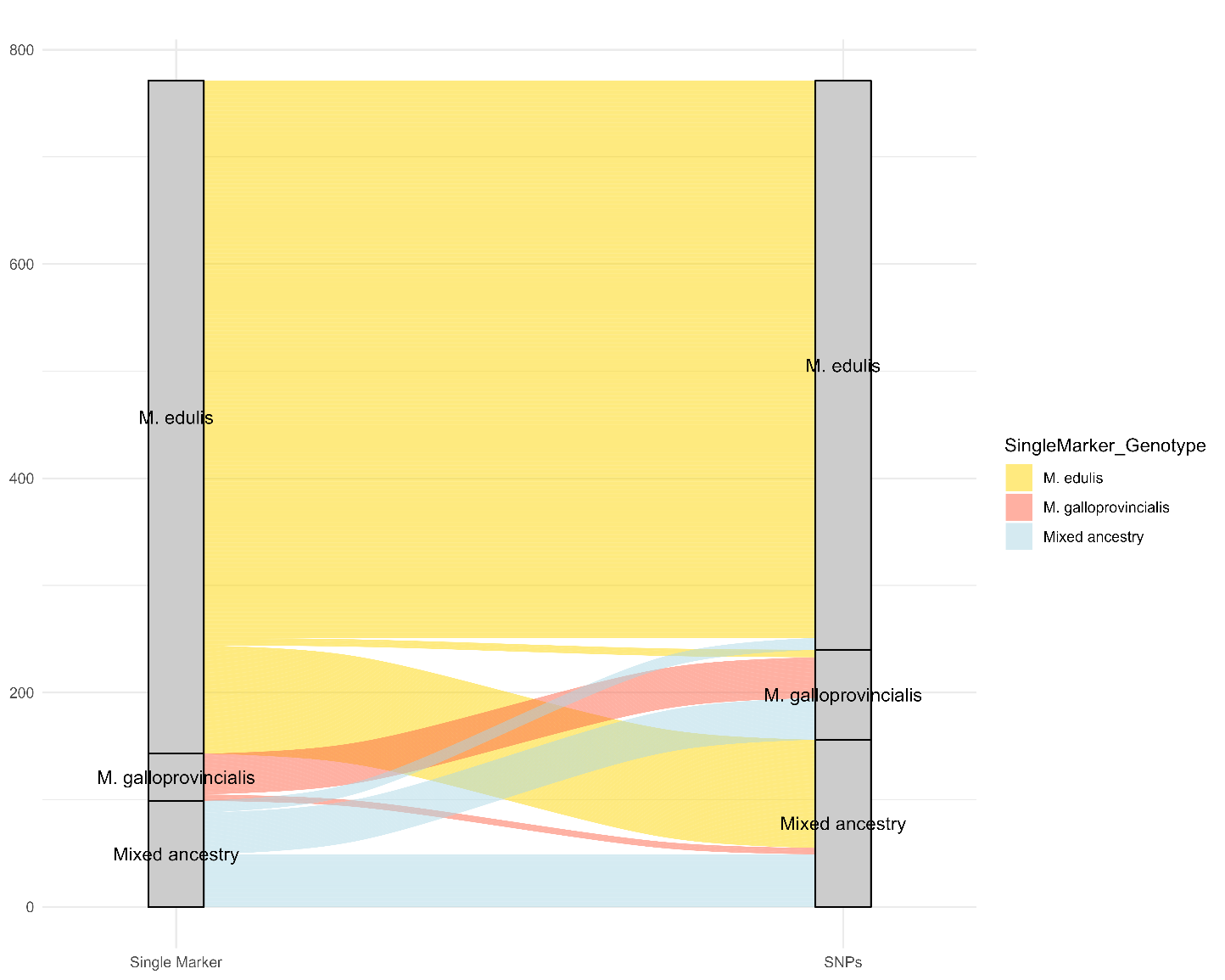


**Figure S1:** Alluvial plot showing the correspondence between genotype assignments obtained using the adhesive protein gene single marker (Inoue et al., 1995) and the SNP panel used in this study (genotype threshold from (Mathiesen et al., 2017). The X-axis represents genotype assignments based on the single marker (left) and the SNPs panel (right), while the Y-axis indicates the total number of genotyped mussels. Colours indicate genotype: yellow for M. edulis, red for M. galloprovincialis, and blue for individuals of mixed ancestry


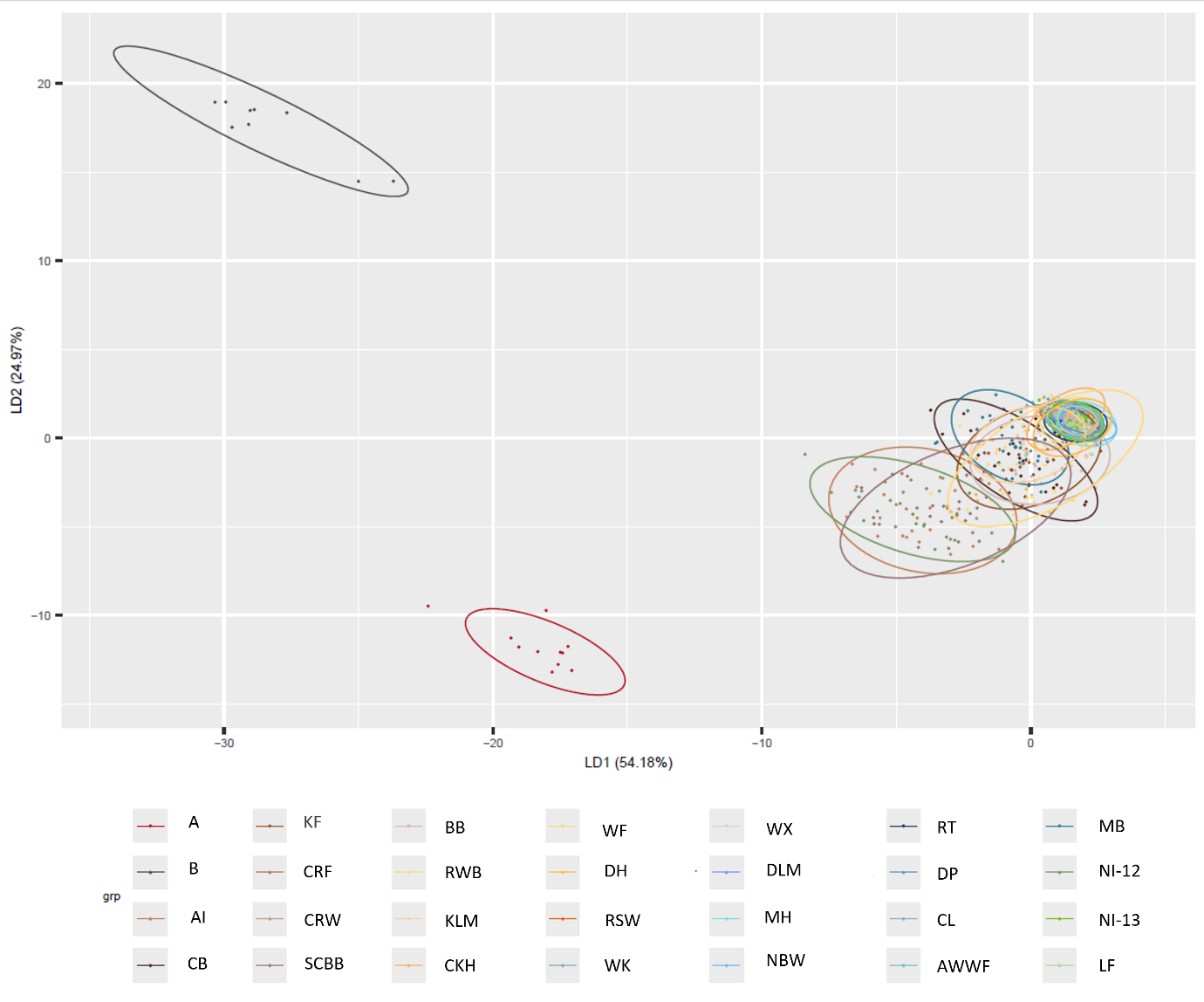


**Figure S2:** DAPC plot of the full dataset, including all Irish, Adriatic and Baltic samples, employing the initial panel of 82 SNP loci resulting from the initial genotyping QC. Brown shades colour indicates sites from the west coast of Ireland, yellow and orange colours from the south coast, blue from the east coast and green from the north coast (Republic of Ireland and Northern Ireland, UK). The proportion of overall variation for each LD is indicated in percentage. Site codes information can be found in Table 1.


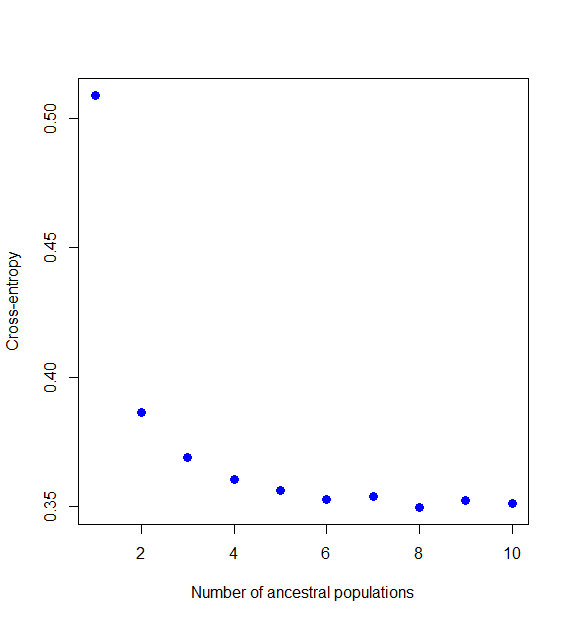


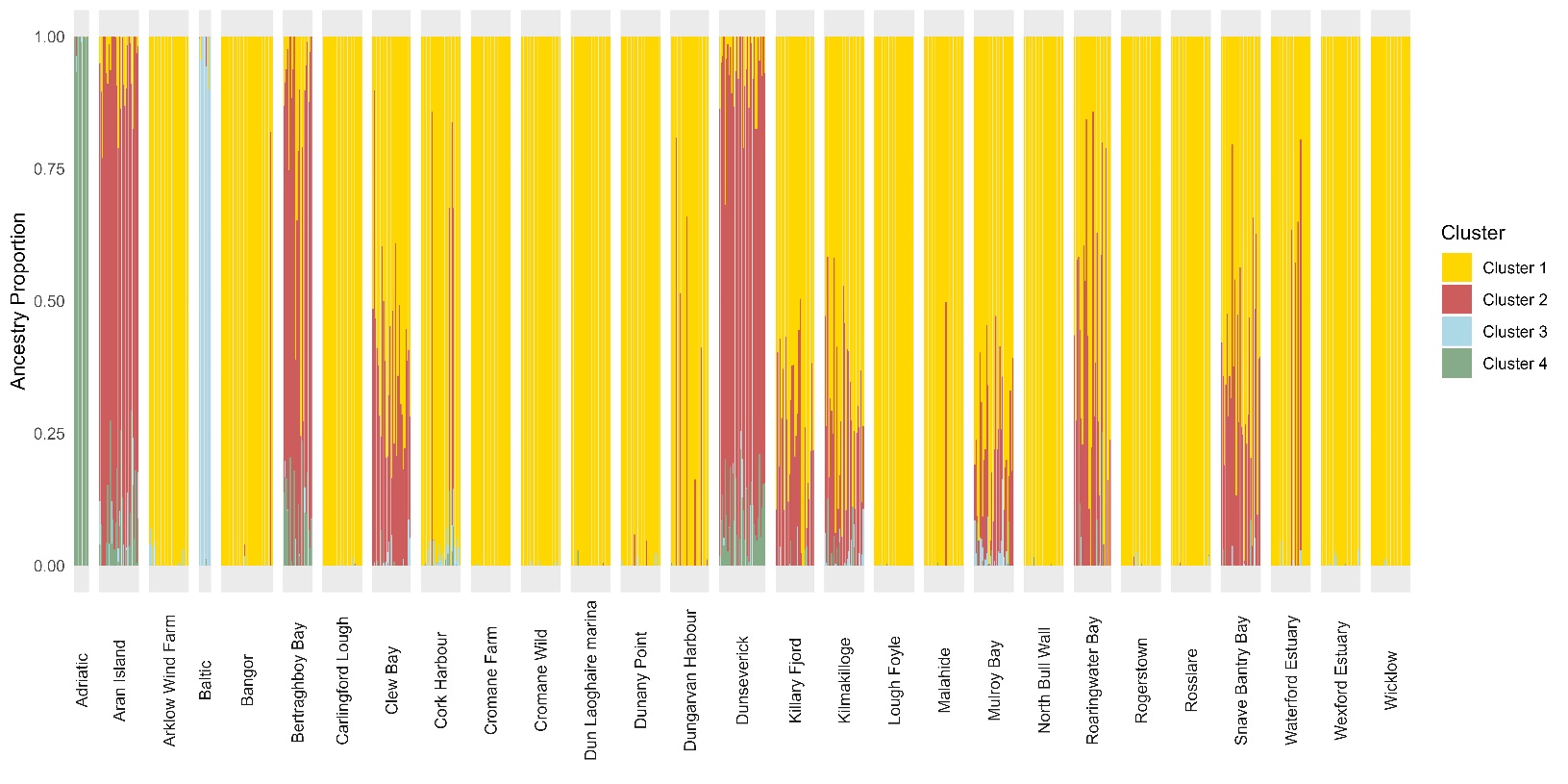


**Figure S3:** Plots from the function sNMF from the R package LEA to explore Admixture proportions of the initial dataset (Irish, Adriatic and Baltic samples, 82 SNP loci). Top figure - Cross-entropy plot indicating four as the optimal number of clusters. Bottom Figure - Admixture plot: each column represents an individual, and individuals are grouped by populations on the X axis. Y axis indicates the Ancestry Proportion (Q value), and columns are coloured proportionally according to the composition of each of the four clusters.


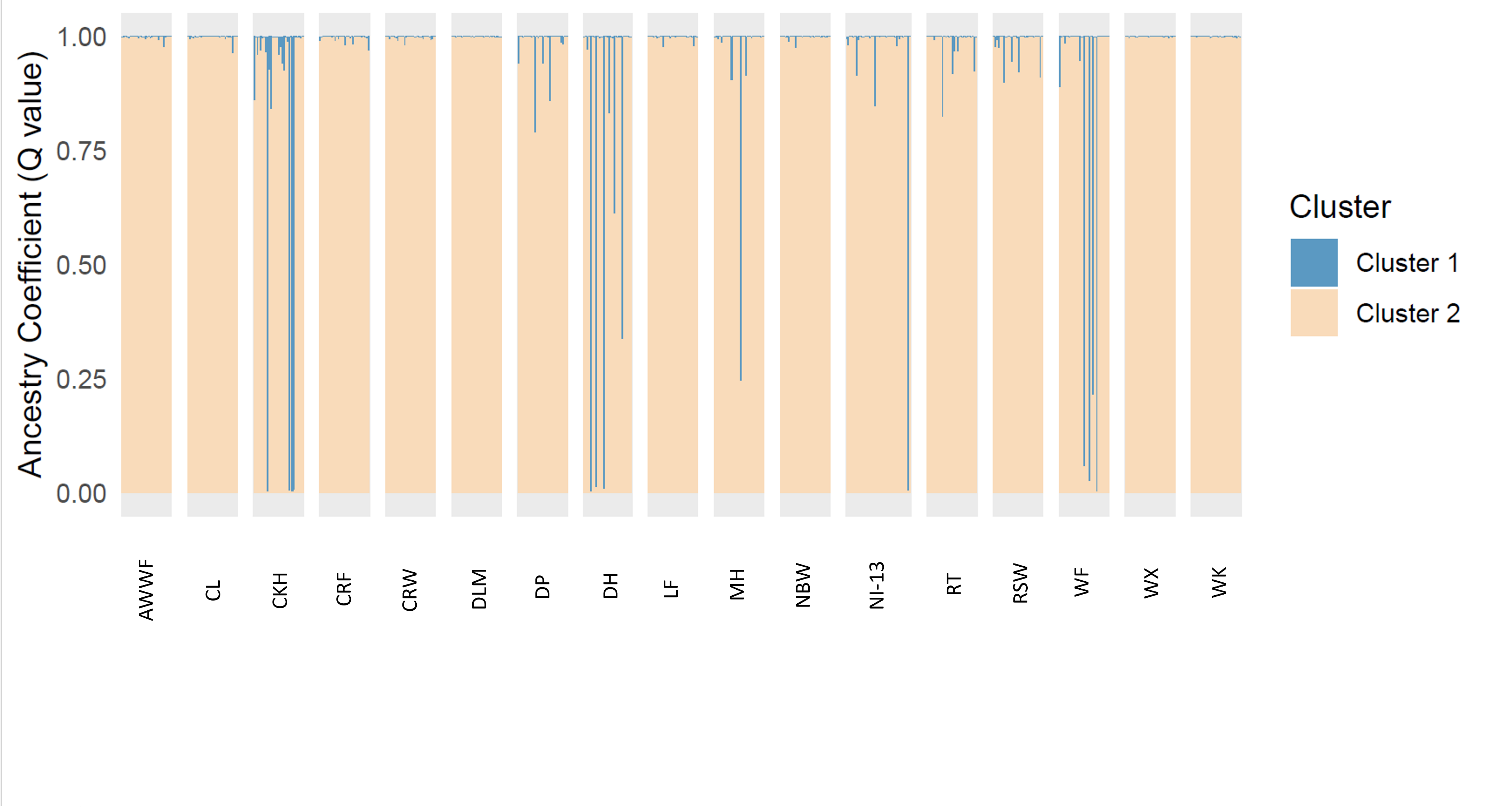


**Figure S4:** Results from STRUCTURE with best K=2 of all Irish populations that on average have <0.2 level of admixture, to investigate the population structure, genetic admixture and ancestry inference of the “pure” Irish M. edulis genotype, employing the panel of 72 SNP loci. Each column represents an individual, and individuals are grouped by populations on the X axis. Y axis indicates the Ancestry Coefficient (Q value), and columns are coloured proportionally according to the composition of each of the two clusters. Site codes information can be found in Table 1.


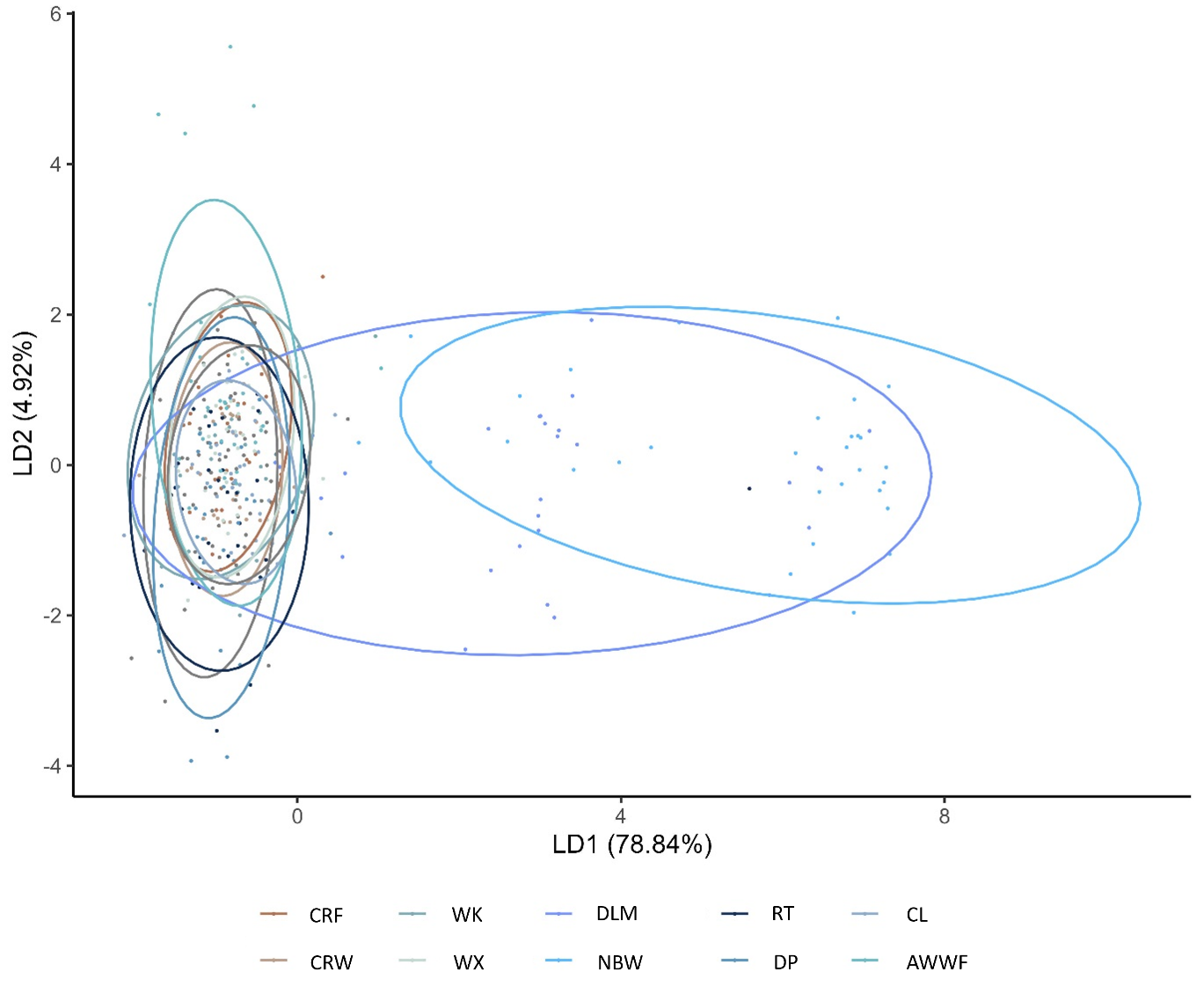


**Figure S5:** DAPC plot of the “pure” Irish M. edulis genotype dataset (admixture proportion <0.2) employing the panel of 72 SNP loci. Brown shades colour indicates sites from the west coast of Ireland, and blue shades colour from the east coast. The proportion of overall variation for each LD is indicated in percentage. Site codes information can be found in Table 1.

.


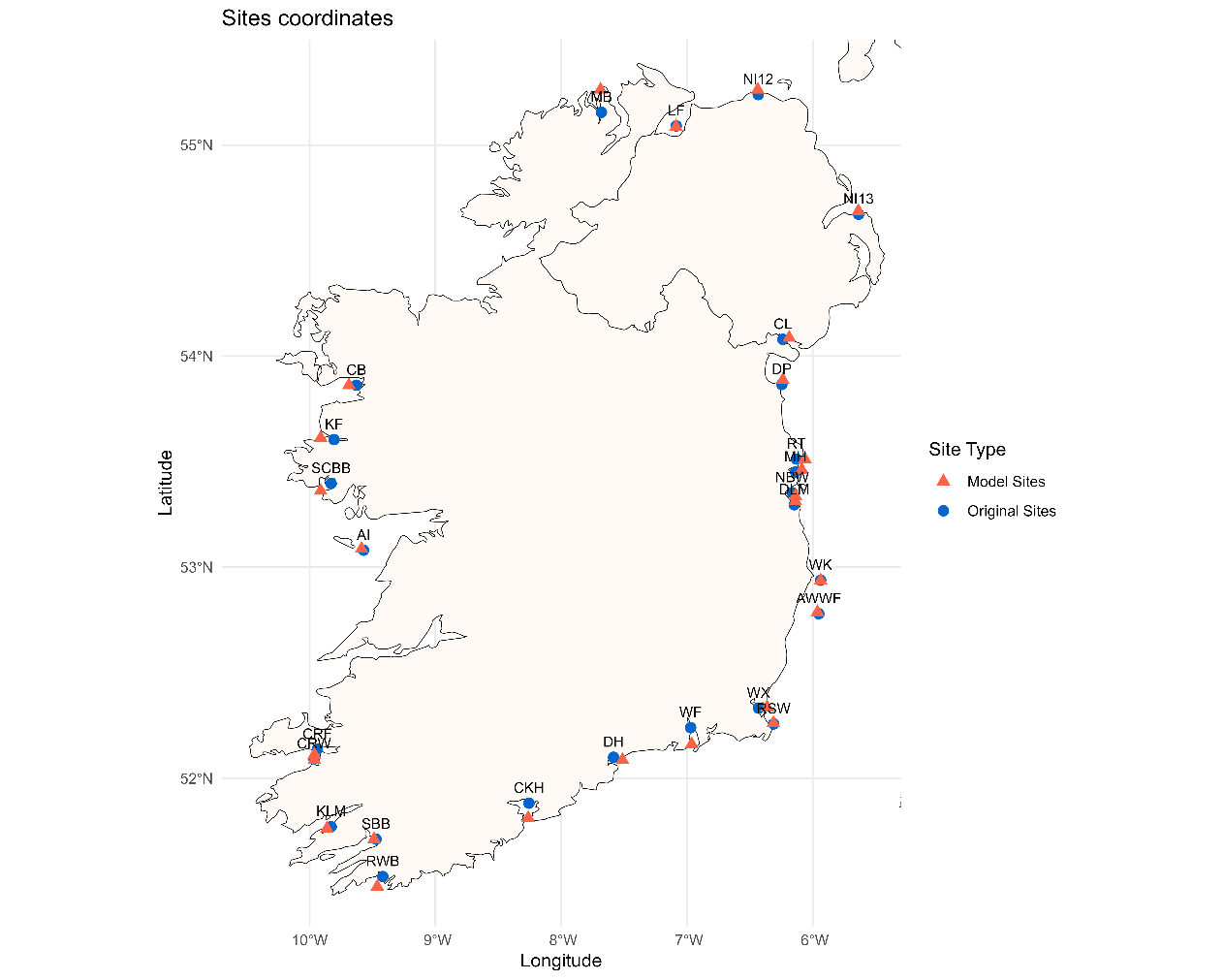


**Figure S6:** Map of sites coordinates. Blue dots are the original sampling sites (as in Table 1), the red triangle are the final sites employed in both the IBD and environmental beta regression models (coordinates in Table S4). Site codes information can be found in Table 1.


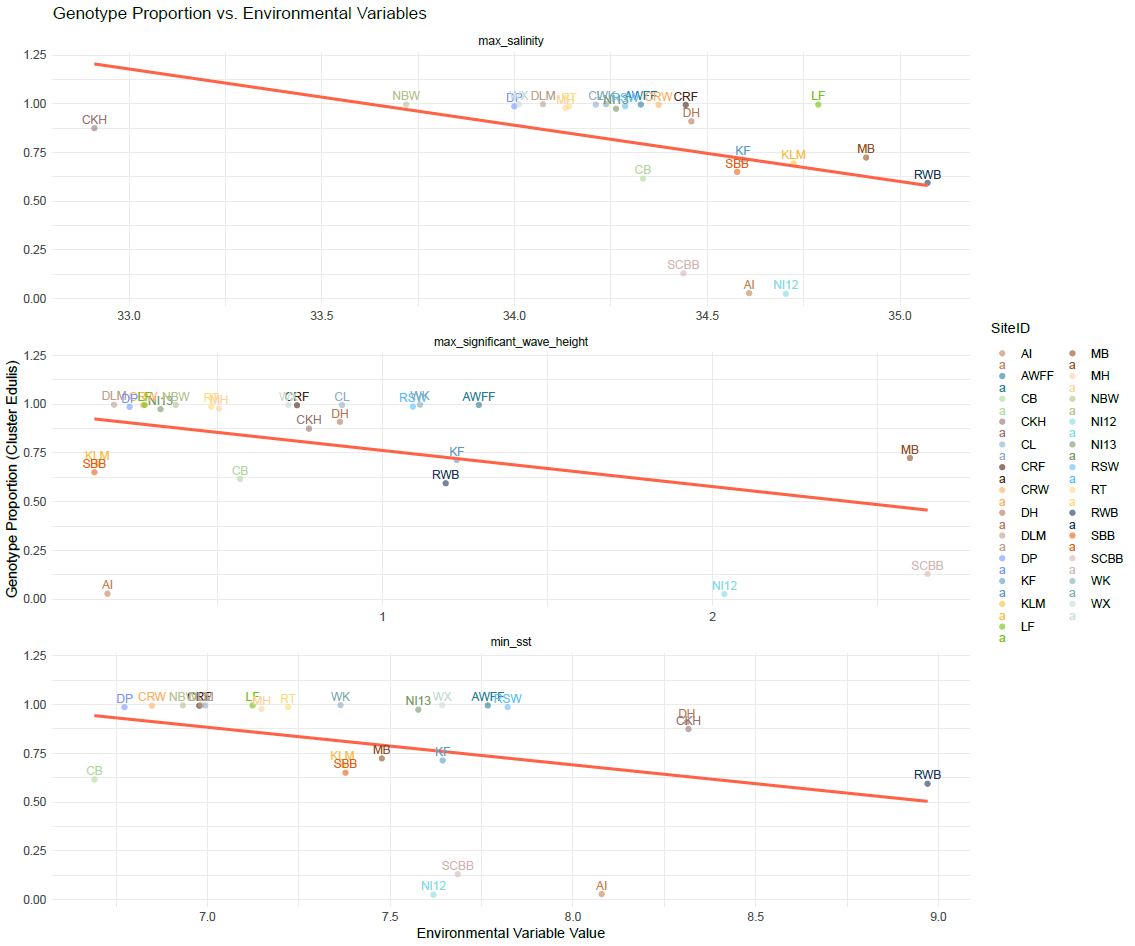


**Figure S7:** Preliminary Beta regression scatterplot of M. edulis genotype proportion (Q values) against environmental variables, excluding Waterford Estuary. Brown shades colour indicates sites from the west coast of Ireland, yellow and orange colours from the south coast, blue from the east coast and green from the north coast (Republic of Ireland and Northern Ireland, UK). Site codes information can be found in Table 1.

**Table S1:** List of SNP loci markers resulting from summary statistics (monomorphic, allele frequencies <0.01, F_is_ >± 0.3) and their inclusion/exclusion from the different datasets used throughout the paper analyses.

| Locus | Monomorphic Markers for Irish samples | Allele freq <0.01 | Fis >+- 0.3 without admix population | Markers excluded from 72 SNPs loci panel | Markers excluded from 63 SNPs loci panel |
| --- | --- | --- | --- | --- | --- |
| 108748_B |  |  |  |  |  |
| Contig17324_GA36A |  |  |  |  | x |
| Contig96364_GA36A |  |  |  |  |  |
| 802-C9777_GA36C_p1086 |  |  |  |  |  |
| 603-mac1 |  |  |  |  |  |
| 37630_A |  | yes |  | x | x |
| Contig44265_GA36C |  |  |  |  |  |
| 209-R_L10-14_mira_C1 |  |  |  |  |  |
| 184-soap_C3118_p4466 |  |  |  |  | x |
| 180-soap_C254_p1675 |  |  |  |  |  |
| 115-gi_384113217 |  |  |  |  |  |
| 129715_A |  |  |  |  | x |
| Contig17424_GA36H |  |  |  |  |  |
| Contig9777_GA36C |  |  |  |  |  |
| 801-C6813_GA36C_p732 |  |  |  |  |  |
| 196-soap_C415_p534 |  |  |  |  |  |
| 124-gi_387154968 |  |  |  |  |  |
| 39506_A | yes | yes |  | x | x |
| Contig4715_GA36B |  |  |  |  |  |
| 155-abyss_C477_p4647 |  |  |  |  |  |
| 152-abyss_C426_p2707 |  |  |  |  |  |
| 604-Efa2_e6 |  |  |  |  | x |
| 138-abyss_C1219_p217 | yes | yes | excluded in the average and SD of the Fis plot | x | x |
| 130534_A |  |  |  |  |  |
| Contig20739_GA36B |  |  |  |  |  |
| 615-gi387155337_p1866 |  |  | yes | x | x |
| 187-soap_C3422_p3286 |  |  |  |  |  |
| 409-gi_223026752 |  |  |  |  |  |
| 142-abyss_C1506_p145 |  |  |  |  |  |
| 93454_A |  |  |  |  |  |
| Contig6231_GA36B |  |  |  |  |  |
| 701-C116849_p267 |  |  |  |  | x |
| 164-abyss_C906_p3592 |  |  |  |  |  |
| 014-Contig16504_pos702_Edu_EU_US |  |  |  |  |  |
| 127-gi_387154976 |  |  |  |  |  |
| 152471_B |  |  |  |  |  |
| Contig23582_GA36B |  |  |  |  |  |
| 111-gi_384113180_emb_HE609053_pos1114_stl_tva | yes | yes |  | x | x |
| 161-abyss_C898_p188 |  |  |  |  |  |
| 117-gi_38635427 |  |  |  |  |  |
| 94351_B |  |  |  |  |  |
| Contig63342_GA36B |  |  |  |  |  |
| 505-abyss_C723_p2319 |  |  |  |  |  |
| 202-L02_mira_C1_p242 |  |  |  |  |  |
| 205543_A |  |  |  |  |  |
| Contig26709_GA36B |  |  |  |  |  |
| 508-soap_C2331_p1911 |  |  |  |  |  |
| 702-gi_223023995 |  |  |  |  |  |
| Contig10081_GA36A |  |  |  |  |  |
| Contig73535_GA36A |  |  |  |  |  |
| 429-gi_261362848_gb_Contig28358_p4108 |  |  |  |  | x |
| 159-abyss_C783_p5373 |  |  |  |  |  |
| 109-gi_380851736_gb_GA |  |  |  |  |  |
| 220088_B |  |  |  |  |  |
| Contig27467_GA36B |  |  |  |  |  |
| 109-gi_380851736_gb_GT |  |  |  |  |  |
| 160-abyss_C783_p5440 |  |  |  |  |  |
| 038-Contig31315_pos1274_Edu_EU_US |  |  |  |  |  |
| 147-abyss_C244_p6092 |  |  |  |  |  |
| Contig10366_GA36G |  |  |  |  |  |
| 148-abyss_C255_p3048 |  |  |  |  | x |
| 428-gi_384575681_emb_HE609049_p1650 |  |  | yes | x | x |
| 162-abyss_C906_p1084 |  |  |  |  |  |
| 24411_A |  |  |  |  |  |
| 502-abyss_C1118_p395 |  |  |  |  |  |
| 206-R_L04_newbler_C0 |  |  |  |  |  |
| 211-R_L22_mira_C11 |  |  |  |  |  |
| 144-abyss_C216_p1703 |  |  |  |  |  |
| Contig14012_GA36H |  |  |  |  |  |
| Contig77511_GA36A |  |  |  |  |  |
| 190-soap_C3470_p5286 |  |  |  |  |  |
| 108-gi_37650308_gb_AJ516731_pos350_stl_tva | yes | yes | excluded in the average and SD of the Fis plot | x | x |
| 106-gi_261362848 |  |  |  |  |  |
| 33513_A | yes | yes |  | x | x |
| Contig401454_GA36A |  |  |  |  |  |
| 200-M_L03_soap_C100 |  |  |  |  |  |
| 703-gi_384113199 |  |  |  |  | x |
| 143-abyss_C1671_p375 |  |  |  |  | x |
| Contig14115_GA36B |  |  |  |  |  |
| 174-H_L1_soap_Contig1865_pos4732_Edu_EU_US | yes | yes | excluded in the average and SD of the Fis plot | x | x |
| 166-soap_C1072_p3271 |  |  |  |  |  |
| 128-gi_387155337 |  |  | yes | x | x |

| **Population** | **Ar** | **Ho** | **He** | **F*is*** |
| --- | --- | --- | --- | --- |
| Aran Island (Inish Meáin) | 1.83 | 0.31 | 0.32 | 0.06 |
| Clew Bay | 1.93 | 0.23 | 0.27 | 0.14 |
| Cromane Farm | 1.32 | 0.07 | 0.07 | 0.09 |
| Cromane Wild | 1.33 | 0.08 | 0.08 | 0.07 |
| Killary Fjord | 1.87 | 0.18 | 0.23 | 0.21 |
| Mulroy Bay | 1.83 | 0.2 | 0.23 | 0.12 |
| Snave Bantry Bay | 1.82 | 0.21 | 0.24 | 0.13 |
| Waterford Estuary | 1.67 | 0.1 | 0.12 | 0.19 |
| Rosslare | 1.39 | 0.07 | 0.08 | 0.11 |
| Wicklow | 1.3 | 0.07 | 0.07 | 0.01 |
| Wexford Harbour | 1.29 | 0.07 | 0.07 | 0.06 |
| Dún Laoghaire Marina | 1.28 | 0.08 | 0.07 | -0.02 |
| Malahide | 1.41 | NaN | 0.1 | 0.08 |
| North Bull Wall | 1.3 | NaN | 0.09 | 0.01 |
| Rogerstown | 1.36 | 0.07 | 0.08 | 0.11 |
| Dunany Point | 1.35 | 0.07 | 0.08 | 0.08 |
| Carlingford Lough | 1.33 | 0.06 | 0.07 | 0.09 |
| Cork Harbour | 1.75 | 0.14 | 0.16 | 0.22 |
| Kilmakilloge | 1.86 | 0.21 | 0.24 | 0.14 |
| Arklow Wind Farm | 1.36 | 0.07 | 0.08 | 0.08 |
| Dunseverick | 1.87 | 0.31 | 0.32 | 0.08 |
| Bangor | 1.5 | 0.08 | 0.09 | 0.14 |
| Dungarvan Harbour | 1.71 | 0.1 | 0.13 | 0.16 |
| Lough Foyle | 1.3 | 0.06 | 0.07 | 0.10 |
| Bertraghboy Bay | 1.88 | 0.28 | 0.32 | 0.18 |
| Roaringwater Bay | 1.86 | 0.2 | 0.26 | 0.18 |

**Table S2:** Top table: Allele richness (Ar), Observed Heterozygosity (Ho), Expected Heterozygosity (He) calculated per populations with diveRsity, and Inbreeding coefficient (Fis) calculated with GenePop rounded to two decimals. Bottom table: average of Ar, Ho, He, and Fis according to genotype ancestry of Irish populations.

| **Genotype** | **Ar** | **Ho** | **He** | **Fis** | **SD-Ar** | **SD-Ho** | **SD-He** | **SD- Fis** |
| --- | --- | --- | --- | --- | --- | --- | --- | --- |
| *M. galloprovincialis* | 1.86 | 0.3 | 0.32 | 0.10 | 0.03 | 0.017 | 0 | 0.07 |
| *M. edulis* | 1.39 | 0.075 | 0.08 | 0.08 | 0.13 | 0.012 | 0.018 | 0.07 |
| Admixed | 1.85 | 0.195 | 0.23 | 0.16 | 0.05 | 0.028 | 0.035 | 0.04 |

**Table S3**: Populations Ne index estimated with MAF<0.02 using RLDNe R package.

| Population | Estimated Ne MAF<0.02 |
| --- | --- |
| Aran Island | 86.6 |
| Clew Bay | 39.8 |
| Cromane Farm | Inf |
| Cromane Wild | 40.9 |
| Killary Fjord | 193.5 |
| Mulroy Bay | 121.5 |
| Snave Bantry Bay | 47.2 |
| Waterford Estuary | 1 |
| Rosslare | 69.3 |
| Wicklow | 514.8 |
| Wexford Estuary | 54.4 |
| Dun Laoghaire Marina | 469.1 |
| Malahide | 13.7 |
| North Bull Wall | Inf |
| Rogerstown | 43.7 |
| Dunany Point | 102.3 |
| Carlingford Lough | Inf |
| Cork Harbour | 0.9 |
| Kilmackillogue | 26.5 |
| Arklow Wind Farm | 30 |
| Dunsevrick | 493.8 |
| Bangor | 3.4 |
| Dungarvan Harbour | 1.2 |
| Lough Foyle | Inf |
| Bertraghboy Bay | 14.2 |
| Roaringwater Bay | 7.8 |

**Table S4:** Coordinates of the sites employed in the different analysis. Latitude and Longitude are the original sampling coordinates. New.lat and new.lon are the latitude and longitude used for retrieving environmental data from the Marine Institute NEATL model and Copernicus Marine Service, as well as to run the IBD analyses. Geodist distance between the original site locations and the new positions calculated using the distGeo function from the geosphere R package

| Location | Region | Latitude | Longitude | new.lat | new.lon | geodist |
| --- | --- | --- | --- | --- | --- | --- |
| Mulroy Bay | North | 55.15 | -7.68 | 55.26 | -7.68 | 11.90 |
| Lough Foyle | North | 55.09 | -7.08 | 55.08 | -7.08 | 0.39 |
| Clew Bay | West | 53.86 | -9.62 | 53.86 | -9.68 | 6.55 |
| Killary Fjord | West | 53.60 | -9.80 | 53.61 | -9.91 | 11.78 |
| Bertraghboy Bay | West | 53.39 | -9.82 | 53.36 | -9.91 | 9.92 |
| Aran Island (Inish Meáin) | West | 53.08 | -9.57 | 53.08 | -9.58 | 1.92 |
| Cromane Wild | Southwest | 52.09 | -9.96 | 52.08 | -9.96 | 0.66 |
| Cromane Farm | Southwest | 52.13 | -9.93 | 52.11 | -9.96 | 3.90 |
| Snave Bantry Bay | Southwest | 51.71 | -9.47 | 51.71 | -9.48 | 1.63 |
| Roaringwater Bay | South | 51.53 | -9.41 | 51.48 | -9.46 | 7.13 |
| Cork Harbour | South | 51.88 | -8.25 | 51.81 | -8.26 | 7.74 |
| Waterford Estuary | South | 52.24 | -6.97 | 52.16 | -6.96 | 8.66 |
| Kilmakilloge | South | 51.77 | -9.82 | 51.76 | -9.86 | 3.77 |
| Dungarvan Harbour | South | 52.10 | -7.58 | 52.08 | -7.51 | 8.06 |
| Rosslare | East | 52.25 | -6.31 | 52.26 | -6.31 | 0.50 |
| Wexford Harbour | East | 52.33 | -6.43 | 52.33 | -6.36 | 7.63 |
| Arklow Wind Farm | East | 52.78 | -5.95 | 52.78 | -5.96 | 1.40 |
| Wicklow | East | 52.93 | -5.93 | 52.93 | -5.93 | 0.14 |
| Dún Laoghaire Marina | East | 53.29 | -6.14 | 53.31 | -6.13 | 2.05 |
| North Bull Wall | East | 53.35 | -6.16 | 53.33 | -6.13 | 3.36 |
| Malahide | East | 53.45 | -6.13 | 53.46 | -6.08 | 5.59 |
| Rogerstown | East | 53.51 | -6.13 | 53.51 | -6.06 | 7.46 |
| Dunany Point | East | 53.86 | -6.24 | 53.88 | -6.23 | 2.56 |
| Carlingford Lough | East | 54.07 | -6.23 | 54.08 | -6.18 | 5.55 |
| Dunseverick | North | 55.23 | -6.43 | 55.26 | -6.43 | 2.65 |
| Bangor | North | 54.67 | -5.63 | 54.68 | -5.63 | 1.72 |

## Reference genome details

LOLA: European Nucleotide Archive, project IDs PRJEB24883; Gene Bank GCA_900618805.1; Gerdol et al., 2020

Pura: GCA_001676915.1; Murgarella et al., 2016: <https://doi.org/10.1371/journal.pone.0151561>

e_carboni: GCA_905397895.1; Corrochano-Fraile et al., 2022

<https://doi.org/10.1186/s12864-022-08575-9>

e_hori: GCA_019925275.2; Regan et al., 2024 <https://doi.org/10.1093/g3journal/jkae138>

All GAL and ITA genomes (GALF1, GALF2, GALF3, GALM1, GALM11, GALM2, GALM3, GALM6, ITAF1, ITAF2, ITAF3, ITAM1, ITAM2, ITAM3): project IDs PRJEB24883; Gerdol et al., 2020

Supplementary Methods S1: Protocol optimization for SNPs genotyping on microfluidic Biomark HD platform

To design the new SNP Type™ Assays for SNP Genotyping on the Dynamic Array™ IFCs, 1000bp sequence flanking the selected SNP (SNP position located approximately in the middle of the 1000bp) was extracted from LOLA *M. galloprovincialis* genome assembly (European Nucleotide Archive, project IDs PRJEB24883; Gene Bank GCA_900618805.1; Gerdol et al., 2020) using the CLC Genomics Workbench v. 21.0.5 software (QIAGEN, Aarhus, Denmark. <https://digitalinsights.qiagen.com/>). To check for unwanted variation in the flanking sequences and ensure variation in the SNP site across the different genotypes, a multiple alignment was performed using BioEdit software v. 7.2.5 (Hall T., 1999). Each SNP extracted sequence was visually checked with a set of 17 reference *Mytilus* genomes: two *M. edulis*, 11 *M. galloprovincialis* and 4 *M. galloprovincialis x M. edulis* (Corrochano-Fraile et al., 2022; Gerdol et al., 2020; Murgarella et al., 2016; Regan et al., 2024). Finally, 91 SNPs were successfully curated, and the flanking sequences were submitted to the Standard BioTools™ D3™ Custom Assay Design team, who designed and manufactured the Locus Specific Primers (LSP), Specific Target Amplification primer (STA) and Allele Specific Primer 1 and 2 (ASP1 and ASP2, respectively).

To ensure high-quality SNPs clustering and assisting manual calling, synthetic target DNA fragments (Oligo Pools, Integrated DNA Technologies) representing both variants at each SNP site were included as positive controls in each run at a final concentration of 0.1 µM.

The SNPs genotyping was carried out using a Biomark™ HD high-throughput qPCR system (Standard BioTools™, South San Francisco, CA, USA), employing the SNP Type™ Assays for SNP Genotyping on the Dynamic Array™ IFCs 96.96 (i.e. screening 96 samples at 96 assays in each run). To ensure good quality amplification, genotype calling and clustering, the following optimization steps were implemented in the protocol. A pre-amplification step was carried out for both the samples and the oligo pools positive controls, following Appendix C of the Standard BioTools™ SNP Genotyping Analysis User Guide (PN 68000098), with the adaptation of 18 cycles. Subsequently, pre-amplified samples were diluted 1:10 with molecular grade water, while pre-amplified oligo pools positive control were diluted 1:1000. For each IFC run, duplicates of the three positive controls oligo pools (XX, XY and YY) and duplicates of No Template Controls (NTC) were included.

To obtain the optimum amplification cycle for each SNP clustering, modifications on the thermal cycle conditions of the SNPtype E 96.96 v1 run script protocol from Standard BioTools™ were made as follows: Thermal Mix step at 70°C for 30 minutes and at 25°C for 10 minutes, Hot Start step at 95°C for 5 minutes followed by 4 touchdown PCR cycles (-1°C between each cycle) in which the denaturation step was at 95°C for 15 seconds, the annealing step at 64-61°C for 45 seconds, and the extension step at 72°C for 15 seconds; the touchdown phase was followed by 10 cycles with denaturation at 95°C for 15 seconds, annealing at 60°C for 45 seconds and extension at 72°C for 15 seconds. Finally, 30 cycles with image acquisition at the end of each cycle included: denaturation at 95°C for 15 seconds, annealing at 60°C for 45 seconds, extension at 72°C for 15 seconds and image acquisition step at 20°C for 30 seconds. With this protocol, it was possible to follow in “real-time” the amplification of the samples and the cluster trajectory.

Supplementary Methods S2: Sea Current Resistance Modelling

This section provides additional methodological details supporting the ocean currents resistance modelling described in the main text.

Details on coordinate bounds, model names, and resolution:

Current data for the area -15⁰ to -3⁰ degrees longitude and 49⁰ to 56⁰ latitude were obtained from the Northeast Atlantic ROMS model (Nagy et al., 2020). These data were combined with current data from the Global Ocean Physics Reanalysis, E.U. Copernicus Marine Service Information (CMEMS). *Marine Data Store* (MDS). (DOI: 10.48670/moi-00021; Accessed on 11-February-2025) to cover the wider Northeast Atlantic (-86⁰ to 58⁰ longitude, 31⁰ to 89⁰ latitude). The resolution of the Global Ocean Physics Reanalysis product is 1/12⁰. Rasters of current data from the two data products were combined using the *disaggregate* and *merge* functions from the raster package in R (Hijmans, 2025) preserving the higher spatial resolution of the Northeast Atlantic ROMS model.

Details on merging rasters, and combining currents:

Rasters of mean monthly current speed and direction were created from the rasters of u-component and v-component velocities using the formulae:

$$Current speed \left( m.s^{-1} \right)=\sqrt{u^{2}+v^{2}}$$

$$Current direction (^{0})=mod(180+\frac{180}{\pi}atan2\left( u,v \right),360)$$

A raster stack was created containing mean monthly current speed and direction using the *stack* function in the raster package in R. The cost of moving passively between adjacent cells of the raster was calculated using the *flow.dispersion* function from the rWind package in R (Fernández-López & Schliep, 2019), including a modification to deal with missing data corresponding to land (Arjona et al., 2020). The function uses the algorithm of Munoz et al., (2004) to calculate cost as directly proportional to the difference between the direction of movement and the direction of the sea current, and inversely proportional to sea current speed. Dispersal was set to passive to disallow movement against sea current flow (direction of movement – sea current direction > 90⁰).

Details on path network results:

Of the 325 possible connections, 148 were calculable (i.e. the least-cost distance was below infinity in at least one direction). The values ranged from 5,258 to 5,897,646 with a mean of 1,579,780 and followed a bi-modal distribution with 117 below 1,870,000 and 31 above 5,370,000.

REFERENCES

Arjona, Y., Fernández-López, J., Navascués, M., Alvarez, N., Nogales, M., & Vargas, P. (2020). Linking seascape with landscape genetics: Oceanic currents favour colonization across the Galápagos Islands by a coastal plant. *Journal of Biogeography*, *47*(12), 2622–2633. https://doi.org/10.1111/jbi.13967

Corrochano-Fraile, A., Davie, A., Carboni, S., & Bekaert, M. (2022). Evidence of multiple genome duplication events in Mytilus evolution. *BMC Genomics*, *23*(1). https://doi.org/10.1186/s12864-022-08575-9

Fernández-López, J., & Schliep, K. (2019). rWind: download, edit and include wind data in ecological and evolutionary analysis. *Ecography*, *42*(4), 804–810. https://doi.org/10.1111/ecog.03730

Gerdol, M., Moreira, R., Cruz, F., Gómez-Garrido, J., Vlasova, A., Rosani, U., Venier, P., Naranjo-Ortiz, M. A., Murgarella, M., Greco, S., Balseiro, P., Corvelo, A., Frias, L., Gut, M., Gabaldón, T., Pallavicini, A., Canchaya, C., Novoa, B., Alioto, T. S., … Figueras, A. (2020). Massive gene presence-absence variation shapes an open pan-genome in the Mediterranean mussel. *Genome Biology*, *21*(1), 275. https://doi.org/10.1186/s13059-020-02180-3

Hall T. (1999). BioEdit: a user-friendly biological sequence alignment editor and analysis program for Windows 95/98/NT. *Nucl. Acids. Symp. Ser.*, *41*, 95–98.

Hijmans, R. (2025). *raster: Geographic Data Analysis and Modeling*.

Inoue, K., Waite, J. H., Matsuoka, M., Odo, S., & Harayama, S. (1995). Interspecific variations in adhesive protein sequences of Mytilus edulis, M. galloprovincialis, and M. trossulus. *The Biological Bulletin*, *189*(3), 370–375. https://doi.org/10.2307/1542155

Mathiesen, S. S., Thyrring, J., Hemmer-Hansen, J., Berge, J., Sukhotin, A., Leopold, P., Bekaert, M., Sejr, M. K., & Nielsen, E. E. (2017). Genetic diversity and connectivity within Mytilus spp. in the subarctic and Arctic. *Evolutionary Applications*, *10*(1), 39–55. https://doi.org/10.1111/eva.12415

Munoz, J., Felicisimo, A. M., Cabezas, F., Burgaz, A. R., & Martinez, I. (2004). Wind as a long-distance dispersal vehicle in the Southern Hemisphere . *Science*, *304*(5674), 1144–1147.

Murgarella, M., Puiu, D., Novoa, B., Figueras, A., Posada, D., & Canchaya, C. (2016). A first insight into the genome of the filter-feeder mussel Mytilus galloprovincialis. *PLoS ONE*, *11*(3). https://doi.org/10.1371/journal.pone.0151561

Nagy, H., Lyons, K., Nolan, G., Cure, M., & Dabrowski, T. (2020). A regional operational model for the North East Atlantic: Model configuration and validation. *Journal of Marine Science and Engineering*, *8*(9), 1–27. https://doi.org/10.3390/jmse8090673

QIAGEN. (n.d.). *CLC Genomics Workbench v. 21.0.5 software*. Https://Digitalinsights.Qiagen.Com/.

Regan, T., Hori, T. S., & Bean, T. P. (2024). A chromosome-scale Mytilus edulis genome assembly for aquaculture, marine ecology, and evolution. *G3: Genes, Genomes, Genetics*, *14*(8). https://doi.org/10.1093/g3journal/jkae138
